# Sfrp Controls Apicobasal Polarity and Oriented Cell Division in Developing Gut Epithelium

**DOI:** 10.1371/journal.pgen.1000427

**Published:** 2009-03-20

**Authors:** Makoto Matsuyama, Shinichi Aizawa, Akihiko Shimono

**Affiliations:** 1 Vertebrate Body Plan, Center for Developmental Biology, RIKEN Kobe, Minatojima-Minami, Chuou-ku, Kobe, Japan; 2 Cancer Science Institute of Singapore, National University of Singapore, Centre for Life Sciences #02-07, Singapore, Singapore; University of California San Francisco, United States of America

## Abstract

Epithelial tubular morphogenesis leading to alteration of organ shape has important physiological consequences. However, little is known regarding the mechanisms that govern epithelial tube morphogenesis. Here, we show that inactivation of *Sfrp1* and *Sfrp2* leads to reduction in fore-stomach length in mouse embryos, which is enhanced in the presence of the *Sfrp5* mutation. In the mono-cell layer of fore-stomach epithelium, cell division is normally oriented along the cephalocaudal axis; in contrast, orientation diverges in the *Sfrps*-deficient fore-stomach. Cell growth and apoptosis are not affected in the *Sfrps*-deficient fore-stomach epithelium. Similarly, cell division orientation in fore-stomach epithelium diverges as a result of inactivation of either *Stbm/Vangl2*, an Fz/PCP component, or *Wnt5a*. These observations indicate that the oriented cell division, which is controlled by the Fz/PCP pathway, is one of essential components in fore-stomach morphogenesis. Additionally, the small intestine epithelium of *Sfrps* compound mutants fails to maintain proper apicobasal polarity; the defect was also observed in *Wnt5a*-inactivated small intestine. In relation to these findings, Sfrp1 physically interacts with Wnt5a and inhibits Wnt5a signaling. We propose that Sfrp regulation of Wnt5a signaling controls oriented cell division and apicobasal polarity in the epithelium of developing gut.

## Author Summary

The gastrointestinal tract is generated from the primitive gut tube during embryogenesis. The primitive gut differentiates regionally along the cephalocaudal axis. Individual regions simultaneously acquire specific morphologies through morphogenetic mechanisms. The regional specification of the gut tube is controlled by cross-talk between the mesenchyme and epithelium. However, the morphogenetic mechanisms governing gut formation remain poorly understood. Secreted Frizzled-related protein (Sfrp) is an inhibitor of the Wnt pathway, members of which are expressed in the developing gut. A deficiency of *Sfrp* genes (*Sfrp1*, *Sfrp2*, and *Sfrp5*) results in reduction of fore-stomach length in mice. During normal fore-stomach formation, cell division is oriented along the cephalocaudal axis; in contrast, reduced fore-stomach length in *Sfrps*-deficient mice is associated with the divergence of oriented cell division in tubular epithelial cells. Thus, oriented cell division is one of the essential components in fore-stomach morphogenesis. In addition, *Sfrps*-deficient small intestine epithelium fails to maintain proper apicobasal polarity. We also found that Wnt5a-inactivation leads to a phenotype similar to that induced by *Sfrps*-deficiency in the developing gut, and that Sfrp1 inhibits Wnt5a signaling. We propose that Sfrp regulation of Wnt5a signaling is required for oriented cell division and that it modulates apicobasal polarity in gut epithelium during organ elongation.

## Introduction

Generation of the gastrointestinal (GI) tract is initiated by formation of the primitive gut tube during embryogenesis. Subsequently, this tube differentiates regionally along the cephalocaudal axis, giving rise to the esophagus, stomach, small intestine and colon, as well as acquiring specific morphologies, which are generated through morphogenetic mechanisms. Regional specification of the gut tube involves interactions between splanchnic mesoderm and endoderm epithelium [1]. However, the morphogenetic mechanisms governing gut formation remain poorly understood.

Wnt family members are secreted glycoproteins that play important roles in controlling tissue patterning, cell fate, cell proliferation and tissue morphogenesis [2] (http://www-leland.stanford.edu/˜rnusse/wntwindow.html). Wnts are classified into two groups [3]. Wnt1 class ligands (e.g., Wnt1, Wnt3a and Wnt8) activate the canonical Wnt/ß-catenin pathway, which stabilizes ß-catenin as a transcriptional regulator in the nucleus [2],[3]. Wnt5a class ligands (e.g., Wnt5a and Wnt11) stimulate non-canonical Wnt pathways, such as the Ca^2+^ and Fz/PCP pathways, through the Frizzled receptor [3]. Although the role of Wnt signaling in the developing gut is ill-defined, a number of Wnts, Fzs, and their inhibitors, especially Sfrps, are expressed in the tissue [4],[5].

Secreted Frizzled-related protein (Sfrp) is a secreted Wnt antagonist that interacts directly with the Wnt ligand [6]. The *Sfrp* gene family, which consists of five members in both the human and mouse genomes, is classified into the *Sfrp1* (Type 1) and *FrzB* subfamilies based on amino acid sequence similarity [6]. *Sfrp1*, *Sfrp2* and *Sfrp5* comprise the *Sfrp1* subfamily (referred to as Type 1 *Sfrps*) [6]. Type 1 Sfrps inhibit the Wnt/ß-catenin pathway *in vitro*. Type 1 Sfrps exhibit characteristics of Wnt inhibition that differ from those of *FrzB* Sfrps (Sfrp3 and Sfrp4), a phenomenon that can probably be attributed to Wnt ligand specificity [7],[8]. Genetic analysis has revealed the functional redundancy of *Sfrp1*, *Sfrp2* and *Sfrp5*; moreover, *Sfrp1/2/5* genetically interact with *Stbm/Vangl2* (also known as *Ltap*), an ortholog of *Drosophila Strabismus/Van Gogh* Fz/PCP core component [9]. These observations suggest a redundant role for Type 1 Sfrps in the regulation of the Wnt/ß-catenin and the Fz/PCP pathways.

The body axis of *Sfrp1/2/5* compound mutants is shortened [9]. This observation suggests that a concomitant shortening of the axial visceral organs, i.e., the GI tract, may occur. Therefore, we focused on the forming gut tube and epithelial morphogenesis. Our results suggest that Sfrp-regulation of Wnt5a signaling is required for the regulation of epithelial cell polarity, oriented cell divisions and apicobasal (AB) polarity, and lengthening of the developing gut.

## Results

### *Sfrp1*, *Sfrp2*, and *Sfrp5* Are Required for Fore-Stomach Morphogenesis

During mouse embryonic development, the primitive gut tube is generated by embryonic day (E) 9. Subsequently, the gut tube develops the organ buds of the lung, stomach, liver and pancreas, which are apparent at E10.5. In the developing gut, *Sfrp1*, *Sfrp2* and *Sfrp5* are regionally expressed along the cephalocaudal axis. At E10.5, *Sfrp1* was expressed in the splanchnic mesoderm from the caudal region of the prospective stomach to the midgut. At E12.5, expression was observed in the mesenchyme of the colon, as well as in the caudal region of the fore-stomach and the small intestine (Figure S1A, D, G, J, N). *Sfrp2* was expressed in the splanchnic mesoderm of the prospective esophagus at E10.5. Later, at E12.5, *Sfrp2* expression expanded to the rostral region of fore-stomach mesenchyme (Figure S1B, E, H, K, N). *Sfrp2* expression was also detected in colon epithelium at this stage (Figure S1L). In addition, *Sfrp5* expression was present in endoderm cells of the presumptive midgut region at E8.75 [10]. *Sfrp5* expression remained in evidence in the midgut endoderm at E9.5, a stage lacking obvious expression of *Sfrp1* and *Sfrp2* in the gut tube (data not shown). During the later stages of E10.5–12.5, *Sfrp5* expression was observed in the duodenum epithelium (Figure S1C, F, I, M, N). Despite expression in the developing gut, no obvious morphological abnormality was identified in the gut of *Sfrp1*, *Sfrp2* and *Sfrp5* single knock-out embryos as far as we examined [9],[11],[12], possibly because, as in other tissues, the long-range effect of an Sfrp as a secreted factor can compensate for the function of other Sfrps in those mutants [9],[12].

In order to establish the redundant role of Sfrp1, Sfrp2 and Sfrp5 in gut formation, the gut tube was examined in *Sfrp1* subfamily compound mutant mice. *Sfrp1* and *Sfrp5* (*Sfrp1−/− Sfrp5−/−*) and *Sfrp2* and *Sfrp5* (*Sfrp2−/− Sfrp5−/−*) double homozygous mutants appeared to be normal in terms of GI tract formation. In contrast, embryos carrying a double homozygous mutation in both *Sfrp1* and *Sfrp2* (*Sfrp1−/− Sfrp2−/−*) displayed severe shortening of the gut tube, e.g., smaller stomach and shorter intestine, at E13.5 (Figure S2B, E). The reduction in the length/size of the gut in the E13.5 *Sfrp1−/− Sfrp2−/−* embryos was enhanced in the presence of an *Sfrp5* heterozygous mutation (*Sfrp1−/− Sfrp2−/− Sfrp5+/−*), which is suggestive of a redundant role for *Sfrp1*, *Sfrp2* and *Sfrp5* in gut formation (Figure S2E, F).

**Figure 1:**
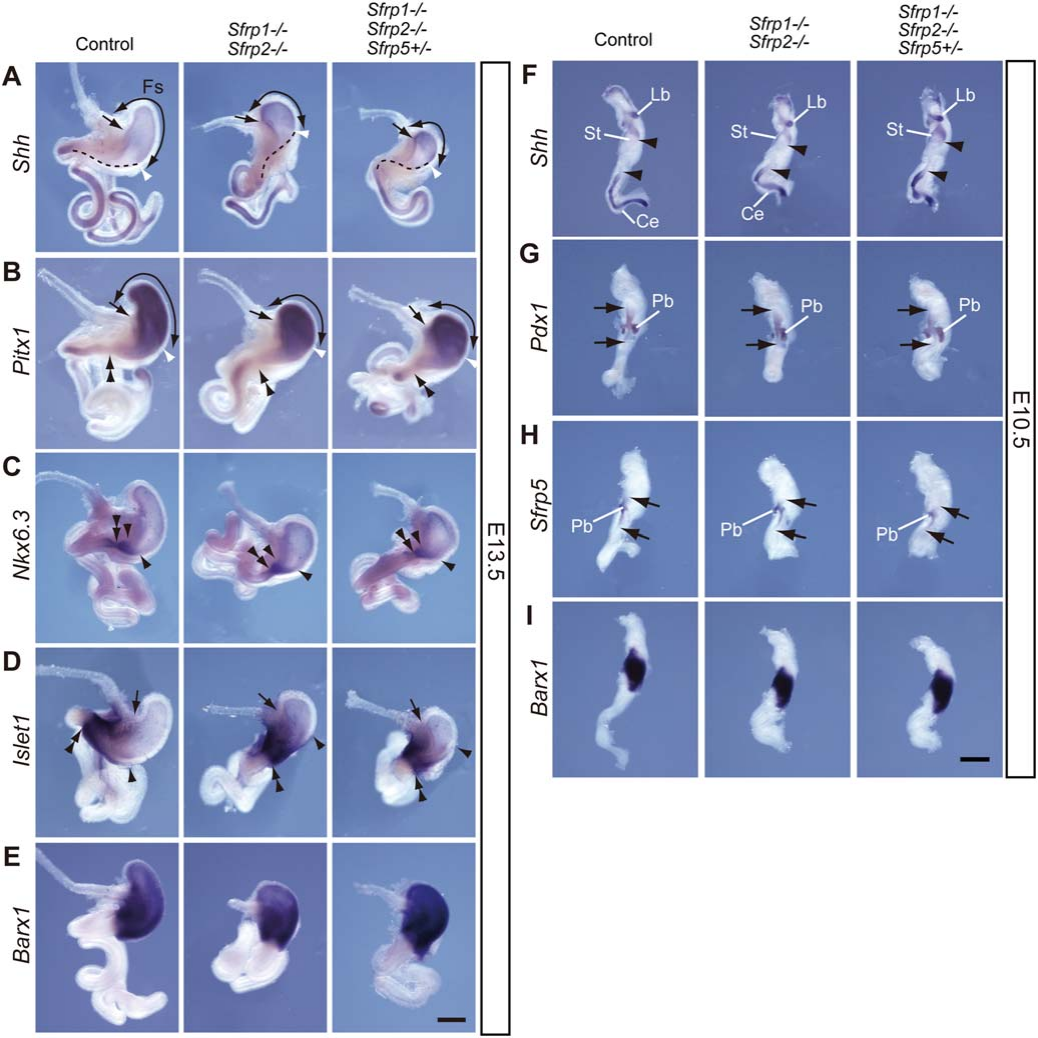
Morphological abnormality in the stomach of *Sfrp1−/− Sfrp2−/−* and *Sfrp1−/− Sfrp2−/− Sfrp5+/−* embryos. (A–E) A small fore-stomach is formed in *Sfrp1−/− Sfrp2−/−* and *Sfrp1−/− Sfrp2−/− Sfrp5+/−* embryos at E13.5, as evidenced by expression of *Shh* (A), *Pitx1* (B), *Nkx6.3* (C), *Islet1* (D) and *Barx1* (E). A broken line depicts *Shh*-negative epithelium along the greater curvature. Curved arrows, an arrow and double arrowheads denote the fore-stomach, the junction of the esophagus and fore-stomach and the pyloric sphincter, respectively. A white arrowhead defines the caudal end of the fore-stomach. A black arrowhead indicates the rostral end of marker gene expression. Fs, fore-stomach. Scale bar: 500 µm. (F–I) A region of presumptive stomach and duodenum is generated normally in *Sfrp1−/− Sfrp2−/−* and *Sfrp1−/− Sfrp2−/− Sfrp5+/−* embryos at E10.5, which is suggested by the expression patterns of *Shh* (F), *Pdx1* (G), *Sfrp5* (H) and *Barx1* (I). The area between the arrowheads identifies a *Shh*-negative region in the gut epithelium (F). Arrows depict the rostral and caudal ends of marker gene expression (G, H). Ce, cecum; Lb, lung bud; Pb, pancreatic bud; St, stomach. Scale bar: 500 µm.

To gain insight into the defect in stomach formation, we examined regional marker expression, *Shh* [13], *Pitx1* [14] and *Nkx6.3* [15], in the epithelium of *Sfrp1−/− Sfrp2−/−* and *Sfrp1−/− Sfrp2−/− Sfrp5+/−* stomachs at E13.5. *Shh* was expressed in fore-stomach and intestinal epithelium of control (Wild-type, *Sfrp1−/−, Sfrp1−/− Sfrp2+/−, Sfrp1−/− Sfrp5+/−* and *Sfrp1−/− Sfrp2+/− Sfrp5+/−*) embryos, while diminished *Shh* expression was evident in the hind-stomach (Figure 1A). *Pitx1* expression was strong in fore-stomach epithelium, but weaker in the hind-stomach (Figure 1B). *Nkx6.3* expression was observed specifically in epithelium extending from the caudal hind-stomach to the duodenum (Figure 1C). All of these epithelial markers were expressed in the stomach of *Sfrp1−/− Sfrp2−/−* and *Sfrp1−/− Sfrp2−/− Sfrp5+/−* embryos; however, *Shh* and *Pitx1* expression exhibited a significant reduction in terms of the size of the fore-stomach (carved arrows in Figure 1A, B). Furthermore, the size reduction of the fore-stomach was enhanced by an *Sfrp5* heterozygous mutation in an *Sfrp1−/− Sfrp2−/−* background (Figure 1A, B). As suggested by the negative region of *Shh* expression (the region indicated by a broken line in Figure 1A), the region marked by *Nkx6.3* expression (Figure 1C) and expression of *Islet1* [16] in hind-stomach mesenchyme (Figure 1D), the hind-stomach at E13.5 appeared to be unaffected by the mutations.

Epithelial specification in the gut is tightly controlled by cross-talk between splanchnic mesoderm and endoderm epithelium [17]. The stomach of compound mutant embryos demonstrated normal expression of *Islet1* (Figure 1D) and *Barx1* [18], which are specific markers for stomach mesenchyme (Figure 1E). The non-glandular stomach of compound mutant embryos was significantly smaller than that of control embryos at E16.5; however, normal characteristic cell types were detected at histological levels in the glandular and non-glandular stomach. The mucosa appeared to be thicker and tightened in *Sfrp1−/− Sfrp2−/− Sfrp5+/−* non-glandular stomach in comparison with control non-glandular stomach (Figure S3).

To determine correlation between reductions of the anterior-posterior (a-p) body axis and abnormal gut formation in *Sfrp1−/− Sfrp2−/−* and *Sfrp1−/− Sfrp2−/− Sfrp5+/−* embryos, we examined the shape of the gut at earlier stages. Reductions in the length of the hindgut and the caudal half of the midgut were already apparent at E10.5. The shortening of the caudal gut tube was closely related to reduction of the a-p body axis in compound mutant embryos at earlier stages [12] (Figure 1F). In contrast, marker analysis of *Shh*, *Pdx1* [19], *Barx1* and *Sfrp5* in *Sfrp1−/− Sfrp2−/−* and *Sfrp1−/− Sfrp2−/− Sfrp5+/−* embryos demonstrated that the regions corresponding to the prospective stomach and duodenum were unaffected at E10.5 (Figure 1F–I). Thus, the region corresponding to the stomach and the duodenum in *Sfrps*-deficient embryos is specified and generated in normal length at E10.5; moreover, organ bud formation is initiated in a manner consistent with that in control embryos. Organ bud formation occurs following the establishment of the a-p body axis; consequently, we concluded that deficiency of Type 1 Sfrps leads to a reduction in the size of the fore-stomach in a manner that is independent of the mechanism that shortens the a-p body axis.

### Sfrps Are Required for Oriented Cell Division in Fore-Stomach Epithelium

Although the stomach region is enlarged at E11.5, one day after the initiation of organ bud formation, the greater curvature of the fore-stomach is not well expanded as observed at later stages. The expansion of the greater curvature becomes obvious from around E12. We measured the size of E12.5 Sfrps-deficient fore-stomach, in order to gain insight into the character of the size reduction defect in the fore-stomach. The length of the greater curvature was greatly reduced in the fore-stomach of *Sfrp1−/− Sfrp2−/− Sfrp5+/−* embryos in comparison with that of control embryos (790±75 µm in control and 406±53 µm in *Sfrp1−/− Sfrp2−/− Sfrp5+/−* fore-stomachs, n = 3, P<0.01). In contrast, the width of the *Sfrp1−/− Sfrp2−/− Sfrp5+/−* stomach was significantly increased at the junction of the fundus and the body (347±33 µm in control and 453±44 µm in *Sfrp1−/− Sfrp2−/− Sfrp5+/−* fore-stomachs, n = 3, P<0.05; Figure S4A–C). Thus, Sfrps deficiency induces lateral expansion of the fore-stomach, which may be suggestive of a defect in morphogenesis.

Since this defect might be associated with an abnormality in epithelium, the histology of Sfrps-deficient fore-stomach epithelium was examined. The cell number per area of epithelium (2000 µm^2^) was slightly increased in the greater curvature of Sfrps-deficient fore-stomachs; however, this observation was statistically insignificant (Figure S4E). Due to the low frequency of multi-nuclei along the apicobasal (AB) axis, the greater curvature epithelium at E12.5 is considered a mono-cell layer in both *Sfrp1−/− Sfrp2−/− Sfrp5+/−* and control fore-stomachs (Figure S4D, F).

**Figure 2:**
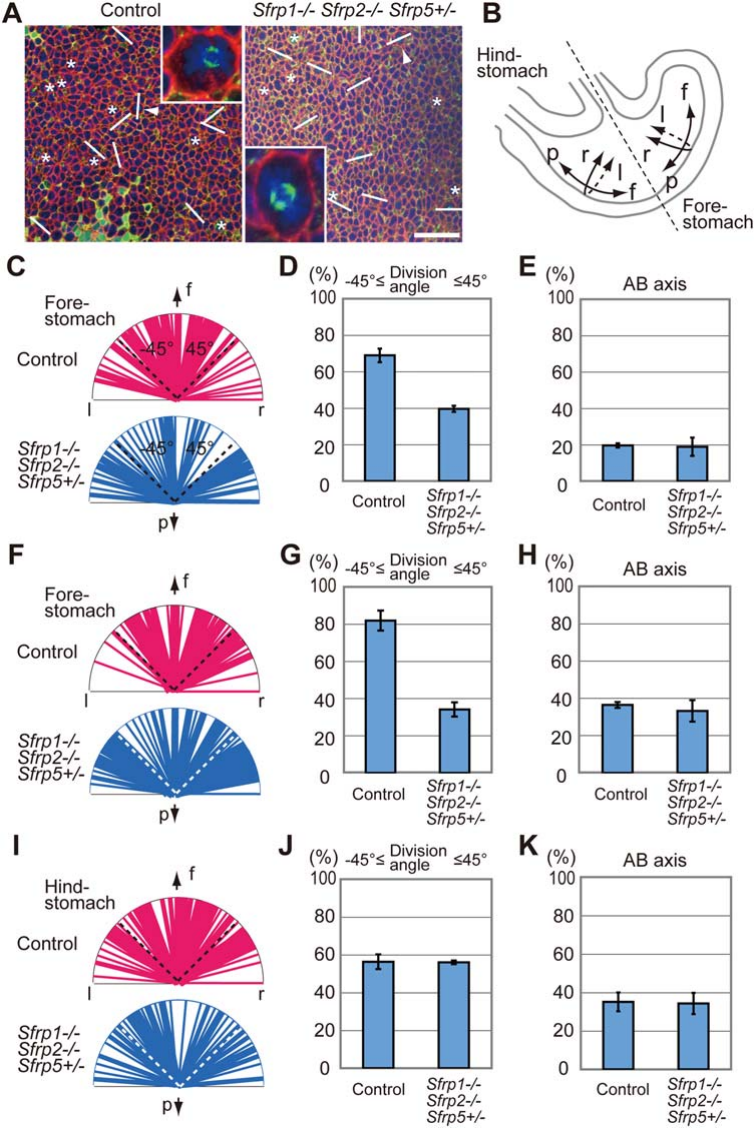
Divergence of cell division orientation in the epithelium of *Sfrps*-deficient fore-stomach. (A) Cell division orientation was visualized with anti-ß1-integrin (red), anti-acetylated α-tubulin (green) and DAPI (blue) staining in the greater curvature epithelium of the fore-stomach of control and *Sfrp1−/− Sfrp2−/− Sfrp5+/−* embryos at E13.5. The top of the confocal image is oriented in the direction of the fundus. Scale bar: 40 µm. An inset shows higher magnification of the mitotic cells indicated by an arrowhead. The cell division axis is indicated by a bar, whereas the vertical axis is denoted by an asterisk. (B) Arrows indicate the direction of the fundus (f), pylorus (p) as well as right (r) and left (l) in the schematic diagram of the stomach at E12.5∼E13.5. (C–H) Cell division orientation converged within ±45° of the cephalocaudal axis in control fore-stomach epithelium, whereas it diverged in *Sfrp1−/− Sfrp2−/− Sfrp5+/−* fore-stomach epithelium at E12.5 (C, D) and E13.5 (F, G). Statistical analysis revealed a significant difference in convergence of cell division orientation along the cephalocaudal axis between control and Sfrps-deficient fore-stomachs at E12.5 (D) and E13.5 (G). In contrast, no significant difference in the frequency of cell division along the AB axis is evident between control and *Sfrp1−/− Sfrp2−/− Sfrp5+/−* fore-stomachs at E12.5 (E) and E13.5 (H). (I, J) Oriented cell division is not observed in hind-stomach epithelium of controls or *Sfrp1−/− Sfrp2−/− Sfrp5+/−* mutants at E13.5. (K) Cell division along the AB axis occurs at a similar frequency in control (35.1±4.93%, n = 3) and *Sfrp1−/− Sfrp2−/− Sfrp5+/−* (34.3±5.5%, n = 3) hind-stomachs at E13.5.

**Figure 3:**
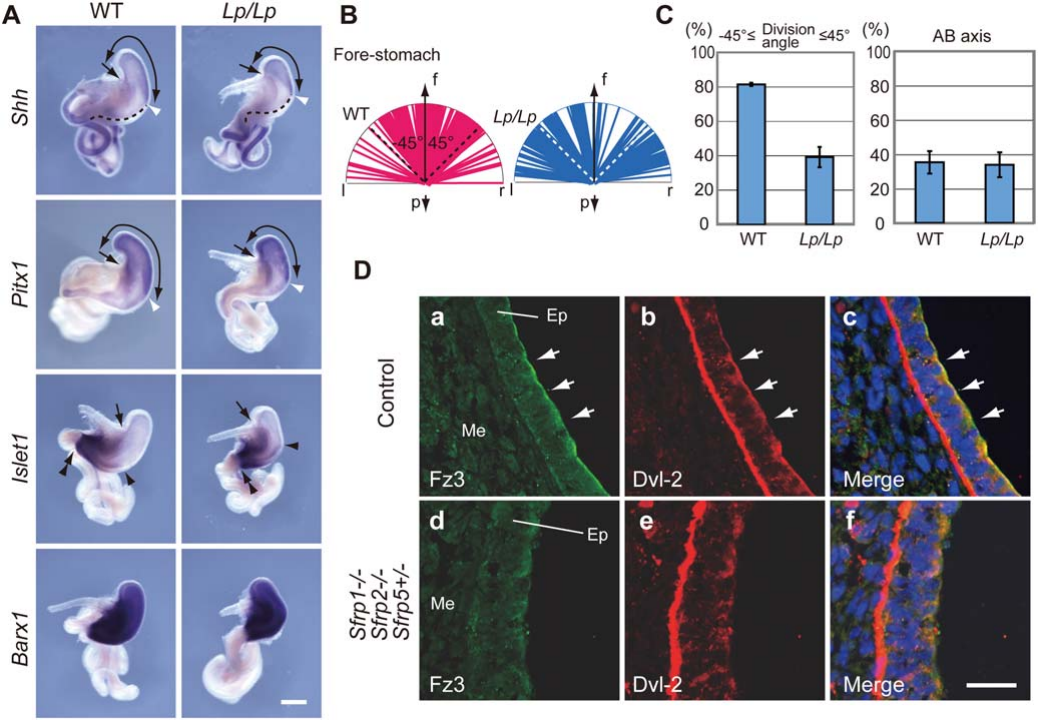
The Fz/PCP pathway is involved in the regulation of oriented cell division in fore-stomach epithelium. (A) The size of the fore-stomach is significantly reduced in *Lp/Lp* embryos at E13.5, as evidenced by *Shh* expression and the higher expression domain of *Pitx1*. *Islet1* and *Barx1* are expressed in mesenchyme of the hind- and whole stomachs. A broken line defines *Shh*-negative epithelium along the greater curvature. Curved arrows, an arrow and double arrowheads denote the fore-stomach, the junction of the esophagus and fore-stomach and the pyloric sphincter, respectively. The white arrowhead identifies the caudal end of the fore-stomach. The black arrowhead indicates the rostral end of marker gene expression. Scale bar: 500 µm. (B) Cell division orientation is diverged in *Lp/Lp* fore-stomach epithelium at E13.5. (C) Statistical analyses revealed a difference between convergence of cell division orientation in controls (81.4±0.96% of 177 cells, n = 3) and *Lp/Lp* fore-stomach (39.2±5.92% of 194 cells, n = 3; P<0.001) epithelium at E13.5 (left). No significant difference in the frequency of cell division along the AB axis is evident between control (35.2±6.51% of 273 cells, n = 3) and *Lp/Lp* (33.8±7.25% of 293 cells, n = 3) fore-stomachs (right). (D) Sfrps involve apical localization of Fz3 and Dvl-2 in fore-stomach epithelium. Fz3 and Dvl-2 are concentrated in the apical region of the epithelium in control E13.5 fore-stomach (Da-c). In contrast, Fz3 and Dvl-2 display diffuse distribution in *Sfrp1−/− Sfrp2−/− Sfrp5+/−* fore-stomach epithelium (Dd-f). Ep, epithelium; Me, mesenchyme. Scale bar: 25 µm.

To elucidate the defect in morphogenesis of the mono-cell epithelial layer in *Sfrp1−/− Sfrp2−/− Sfrp5+/−* fore-stomachs at E12.5, we examined oriented cell division in the greater curvature. The basolateral cellular membrane, microtubule spindles and chromosomes were visualized with anti-ß1-integrin antibody, anti-acetylated α-tubulin antibody [20] and DAPI (4′, 6′-diamidino-2-phenylindole hydrochloride) staining, respectively (Figure 2A, B). The staining of E12.5 fore-stomach epithelium revealed cell division in approximately 3% of cells in the greater curvature of the fore-stomach (3.64±0.32% in control and 3.27±1.12% in *Sfrp1−/− Sfrp2−/− Sfrp5+/−* fore-stomach, n = 3). Approximately 20% of the cell division axis was oriented along the AB axis in both control and Sfrps-deficient fore-stomach epithelium (19.6±1.18% of 204 cells in three controls and 18.9±5.06% of 196 cells in three *Sfrp1−/− Sfrp2−/− Sfrp5+/−* mutants; Figure 2E). Approximately 80% of cell divisions occurred within the horizontal plane of the epithelium, with significant convergence within ±45° of the cephalocaudal axis along the fundus to the pylorus in controls (68.9±3.69% of 164 horizontal mitotic cells in three fore-stomachs; Figure 2C, D). In contrast, oriented cell division was not apparent in the fore-stomachs of *Sfrp1−/− Sfrp2−/− Sfrp5+/−* mutants (39.6±1.71% of 159 horizontal mitotic cells in three fore-stomachs; Figure 3C, D) (P<0.001).

The distinctive abnormality in oriented cell division was maintained at E13.5. Approximately 35% of the cell division axis was oriented along the AB axis in control and *Sfrp1−/− Sfrp2−/− Sfrp5+/−* fore-stomachs (36.5±1.56% of 416 cells in four control and 33.3±5.78% of 465 cells in four *Sfrp1−/− Sfrp2−/− Sfrp5+/−* fore-stomachs; Figure 2H). In the remaining mitotic cells, the orientation of cell division in the horizontal plane displayed convergence within ±45° of the cephalocaudal axis in control fore-stomach epithelium (81.6±5.37% of 266 horizontal mitotic cells in four fore-stomachs; Figure 2H, G). However, cell division orientation diverged markedly in *Sfrp1−/− Sfrp2−/− Sfrp5+/−* fore-stomachs (34.0±3.78% of 312 horizontal mitotic cells in four fore-stomachs, P<0.0001; Figure 2F, G). Oriented cell division was not observed along the cephalocaudal axis of the greater curvature of hind-stomachs of either control or *Sfrp1−/− Sfrp2−/− Sfrp5+/−* embryos (56.3±3.9% of 135 horizontal mitotic cells in three control and 56.0±0.9% of 134 horizontal mitotic cells in three *Sfrp1−/− Sfrp2−/− Sfrp5+/−* hind-stomachs; Figure 2I–K). Hence, these observations suggest that Type 1 Sfrps are required for oriented cell division in the fore-stomach.

Cell proliferation and apoptosis ratios were also examined in *Sfrp1−/− Sfrp2−/− Sfrp5+/−* fore-stomachs. No difference was detected in cell proliferation at E13.5 (14.5±2.5 and 15.4±2.9 phospho-Histone H3-positive cells in 1×10^5^ µm^3^ of control and *Sfrp1−/− Sfrp2−/− Sfrp5+/−* epithelium, respectively; n = 2). The TUNEL assay detected less than 1 apoptotic cell per section of fore-stomach in control and *Sfrps*-deficient embryos; thus, no observations were possible. Similarly, total epithelial cell number in the fore- and hind-stomachs of compound mutant embryos was identical to that in control embryos (Figure S5C, D); however, cell number per area of the greater curvature epithelium (2000 µm^2^) was slightly increased (approximately 27%) in *Sfrp1−/− Sfrp2−/− Sfrp5+/−* fore-stomachs at E13.5 (n = 4, P = 0.0001; Figure S5A, B, E, F).

Since cell density in the epithelium of *Sfrps*-deficient fore-stomachs appeared to be increased in comparison with the controls, we examined AB polarity. Sub-cellular distribution of marker proteins (e.g., atypical Protein Kinase C (aPKC), ß1-integrin, E-cadherin and F-actin) [21]–[23] in *Sfrp1−/− Sfrp2−/− Sfrp5+/−* fore-stomachs was identical to localization in control fore-stomachs (data not shown). However, the distribution patterns did not suggest a strong establishment of AB polarity even in the controls. Thus, these observations suggest that the defect of cell division orientation is associated with fore-stomach morphogenesis phenotype.

### Fz/PCP Pathway Modulates Epithelial Oriented Cell Division in the Fore-Stomach

We next examined which Wnt pathway is regulated by Sfrps in the fore-stomach, since Sfrps regulate the Wnt/ß-catenin and the Fz/PCP pathways [9]. The Wnt/ß-catenin pathway is highly activated in fore-stomach epithelium at E13.5, as evidenced by TOPGAL reporter activity [24] (Figure S6A, B). The activity levels were not altered in fore-stomach epithelium of *Sfrp1−/− Sfrp2−/− Sfrp5+/−* embryos in comparison with that in control embryos. In contrast, TOPGAL activity, which was markedly diminished at the boundary of the control fore- and hind-stomachs, extended into the hind-stomach region in *Sfrp1−/− Sfrp2−/− Sfrp5+/−* embryos (Figure S6C, D). However, as shown by FoxA2 [25] and Sox2 [26] protein expression, no significant patterning defect was observed at the junction of fore- and hind-stomach epithelium (Figure S6E–J). Moreover, the TOPGAL activation pattern in *Sfrp1−/− Sfrp2−/− Sfrp5+/−* stomach was not correlated with the defect observed in fore-stomach morphogenesis.

We also examined fore-stomach morphogenesis in *Lp/Lp* embryos carrying mutations in *Stbm/Vangl2,* a component of the Fz/PCP pathway, because *Type 1 Sfrps* genetically interact with *Stbm/Vangl2* [9]. *Stbm/Vangl*2 is expressed in stomach epithelium [27]. Marker analysis revealed that fore-stomach size/length was greatly reduced in E13.5 *Lp/Lp* embryos (Figure 3A). In addition, a morphological defect was associated with divergence of cell division orientation in the epithelium of the greater curvature epithelium (Figure 3B, C, also see Figure 2B). These observations suggest that the Fz/PCP pathway modulates lengthening of the fore-stomach during oriented cell division.

These data present the possibility that dys-regulation of the Fz/PCP pathway perturbs oriented cell division in *Sfrps*-deficient fore-stomach epithelium. To investigate whether the Fz/PCP pathway is affected in *Sfrps*-deficient fore-stomach, we examined the sub-cellular distribution of Frizzled3 (Fz3) and Dishevelled-2 (Dvl-2) [28],[29]. Proper sub-cellular distribution of Fz/PCP pathway components is essential for the pathway activity [30]. Fz3 and Fz6 are mammalian homologues of the *Drosophila* Fz receptor in the Fz/PCP pathway. Fz3 localized to the apical surface of epithelial cells in control greater curvature at E13.5; in addition, local enrichment of the protein at the site of cell-cell adhesion was not observed. Dvl-2 co-localized with Fz3 in the apical region of the fore-stomach epithelium (arrowheads in Figure 3Da-c). Co-localization of Fz3 and Dvl-2 was less apparent in the apical surface of hind-stomach epithelium (data not shown). Dvl-2 expression, which was also observed in the basal side of the epithelium, overlapped with that of ß1-integrin (Figure 3Db; data not shown). Significantly, Fz3 and Dvl-2 displayed diffuse distribution in the middle of the greater curvature of the *Sfrps*-deficient fore-stomachs (n = 3; Figure 3Dd-f). Thus, this finding indicates that the Fz/PCP pathway is affected in *Sfrps*-deficient fore-stomach. Moreover, Sfrp regulation of the Fz/PCP pathway appears to be correlated with the defect observed in fore-stomach morphogenesis.

### Cell Division Orientation in *Wnt5a−/−* Fore-Stomach Epithelium

Wnt signaling inhibition by Sfrp usually involves an associating Wnt ligand [6]. The following observations suggest that Wnt5a is inhibited by Sfrps during fore-stomach morphogenesis: *Wnt5a*, a typical non-canonical Wnt ligand gene, is expressed in fore-stomach mesenchyme, where defects of the Sfrps-deficient stomach were found; *Wnt5a* and Type1 *Sfrps* genetically interact with *Stbm/Vangl2* to regulate the Fz/PCP pathway [9],[31].

**Figure 4:**
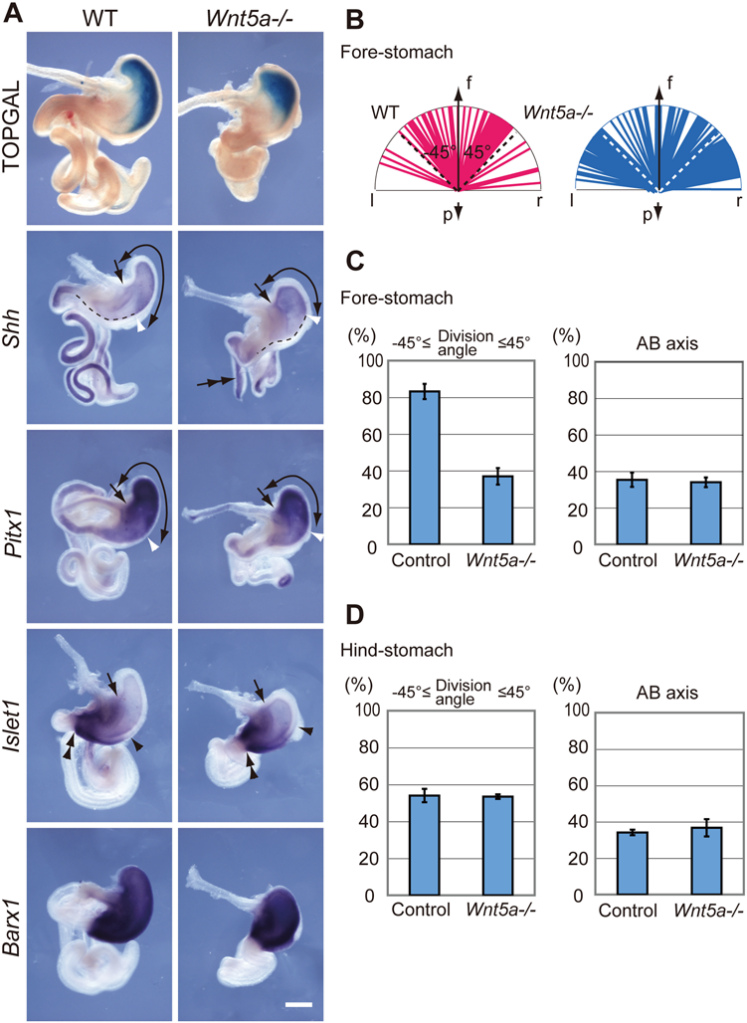
*Wnt5a* homozygous mutations lead to the similar phenotype of *Sfrps*-deficient fore-stomach. (A) *Wnt5a* inactivation results in defective fore-stomach formation at E13.5. TOPGAL activity is unaltered in *Wnt5a−/−* fore-stomach epithelium; however, the size of the fore-stomach is significantly reduced in *Wnt5a −/−* embryos, as evidenced by *Shh* expression and the higher expression domain of *Pitx1*. *Islet1* and *Barx1* are expressed in the mesenchyme of the hind- and whole stomachs. A broken line defines *Shh*-negative epithelium along the greater curvature. Curved arrows, an arrow and double arrowheads denote the fore-stomach, the junction of the esophagus and fore-stomach and the pyloric sphincter, respectively. The white arrowhead identifies the caudal end of the fore-stomach. The black arrowhead indicates the rostral end of marker gene expression. Scale bar: 500 µm. (B) Cell division orientation is diverged in *Wnt5a-*inactivated fore-stomach epithelium at E13.5. (C, D) Statistical analyses revealed a difference between convergence of cell division orientation in control and *Wnt5a−*/*−* fore-stomach epithelia (C, left). Oriented cell division is absent in control and *Wnt5a−*/*−* hind-stomach epithelia (D, left). A significant difference in the frequency of cell division along the AB axis is not evident in either the fore-stomach of the control and *Wnt5a−*/*−* or the corresponding hind-stomachs (right in C and D).

To address the possibility that Wnt5a is an inhibitory target of Sfrps in the fore-stomach, fore-stomach phenotype in Wnt5a homozygous (*Wnt5a−/−*) mutant embryos was surveyed. Significantly, the *Wnt5a−/−*gut displayed similarities to the *Sfrps*-deficient gut, with the exception of ectopic branching of the small intestine (Figure 4A, double arrows in the panel depicting *Shh* expression). First, fore-stomach formation was defective in the *Wnt5a−/−* embryos at E13.5 (Figure 4A). Hind-stomach formation was less affected in the E13.5 *Wnt5a−/−* embryos, although the hind-stomach appeared to be affected and was smaller at later stages, such as E16.5 (data not shown). Second, canonical Wnt/ß-catenin signaling was not altered in that region as evidenced by TOPGAL activity, a reporter of the Wnt pathway [24] (Figure 4A). Third, fore-stomach malformation was associated with divergence of cell division orientation in the greater curvature epithelium (Figure 4B, C, also see Figure 2B). Oriented cell division in the horizontal plane of the greater curvature of the fore-stomach, which was evident in the controls, was not obvious in the *Wnt5a−/−* mutants (Figure 4B).

Statistical analyses revealed a significant difference between convergence of cell division orientation in the control (83.2±4.14% of 232 cells, n = 3) and *Wnt5a−*/*−* fore-stomach epithelia (37.0±4.48% of 257 cells, n = 3, P<0.0001; Figure 4C, left). The cell proliferation ratio determined with anti-phospho-Histone H3 staining was not altered in *Wnt5a−/−* fore-stomach epithelium (18.3±0.2 positive cells in1×10^5^ µm^3^, n = 3) in comparison with control fore-stomach epithelium (18.7±1.0 positive cells in1×10^5^ µm^3^, n = 3). Oriented cell division was not observed in either control (54.0±3.59% of 137 cells, n = 3) or *Wnt5a−*/*−* hind-stomach epithelium (53.4±1.22% of 131 cells, n = 3; Figure 4D). No significant difference in the frequency of cell division along the AB axis was detected between control and *Wnt5a−*/*−* fore-stomachs (35.4±3.91% and 34.0±2.65% of 359 and 389 cells, respectively, n = 3); a similar situation was apparent with respect to corresponding hind-stomachs (34.1±1.54% and 36.7±4.76%, respectively, n = 3; Figure 4C, D, right). Thus, oriented cell division is disrupted in the epithelium of *Wnt5a−*/*−* fore-stomachs as well as in the *Sfrps*-deficient fore-stomach.

### Sfrps Are Involved in AB Polarity in Small Intestine Epithelium

In addition to a defect in fore-stomach morphogenesis, the intestine was shortened substantially in the *Sfrps*-deficient embryos in association with shortening of the anterior-posterior (a-p) body axis (Figure S7). Moreover, the observed reduction in the length of the intestine in E13.5 *Sfrp1−/− Sfrp2−/−* embryos was enhanced in the presence of an *Sfrp5* heterozygous mutation (*Sfrp1−/− Sfrp2−/− Sfrp5+/−*) (Figure S7F). Thus, cell migration associated with a-p axis elongation may be involved in gut morphogenesis. The small intestine was remarkably shortened in *Sfrp1−/− Sfrp2−/−* and *Sfrp1−/− Sfrp2−/− Sfrp5+/−* embryos at E13.5; therefore, *Sfrps*-deficient gut tubes were examined to determine the effect of these molecules on regionalization of the small intestine. *Cdx2* is expressed in the epithelium of the small and large intestines [32]. In the gut derived from *Sfrp1−/− Sfrp2−/−*, *Sfrp1−/− Sfrp2−/− Sfrp5+/−* and control embryos, the rostral boundary of *Cdx2* expression was observed at the pyloric sphincter, a junction between the stomach and the duodenum (Figure S7A, arrow**)**. *Hoxa4* is expressed in the mesenchyme from the prospective duodenum to a portion of the ileum (rostral small intestine) in control embryos [33], whereas *Sfrp5* is expressed in the epithelium (Figure S7B, C). *Wnt5a* and *Hoxc6*, which are marker genes for the caudal small intestine, are expressed in the mesenchyme (Figure S7D, data not shown) [34],[35]. The expression of these markers was indicative of the regionalization of the small intestine in *Sfrp1−/− Sfrp2−/−* and *Sfrp1−/− Sfrp2−/− Sfrp5+/−* embryos. Furthermore, *Hoxd13* expression was observed in the caudal large intestine of *Sfrps*-deficient and control embryos (Figure S7E). Thus, the expression of these markers indicates that (Type 1) Sfrps do not affect regional specification of the gut tube at E13.5.

**Figure 5:**
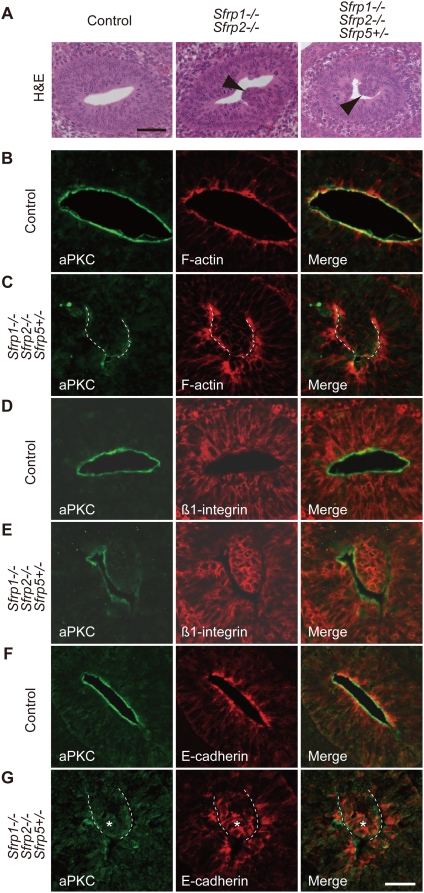
Defective epithelial AB polarity in *Sfrps-*deficient small intestine. (A) Histological (H&E) staining revealed a clump of epithelial cells in the *Sfrp1−/− Sfrp2−/−* and *Sfrp1−/− Sfrp2−/− Sfrp5+/−* small intestine at E13.5 (arrowhead). Scale bar: 50 µm. (B–G) Epithelial AB polarity is disrupted in the *Sfrp1−/− Sfrp2−/− Sfrp5+/−* small intestine (C, E, G) in comparison to the control small intestine (B, D, F), as evidenced by the sub-cellular distribution patterns of activated aPKC (B–G in green), F-actin (B, C in red), ß1-integrin (D, E in red) and E-cadherin (F, G in red). Scale bar: 25 µm.

Interestingly, the small intestine of *Sfrp1−/− Sfrp2−/−* and *Sfrp1−/− Sfrp2−/− Sfrp5+/−* embryos at E13.5 displayed cell clumps, which disrupted the internal surface of the epithelial tube (Figure 5A, arrowhead). The clump of epithelial cells occurred continuously from the jejunum to the ileum. In control embryos at E13.5, the region corresponding to the prospective jejunum and ileum within the small intestine exhibited a smooth apical surface (Figure 5A). In contrast, cell clumps were not obvious in the Sfrps-deficient small intestine at E16.5. It is possible that an increase in cell proliferation may contribute to the generation of cell clumps within the epithelium. However, a BrdU incorporation assay failed to detect an increase in cell proliferation rates in the clumps as well as the entire gut epithelium (36.1±3.38% of total 1030 control epithelium cells, n = 3; 33.6±4.63% of total 1265 *Sfrp1−/− Sfrp2−/− Sfrp5+/−*epithelium cells, n = 3) (Figure S8A–C). In addition, cell density was not significantly increased in the epithelium of *Sfrp1−/− Sfrp2−/− Sfrp5+/−* small intestine (Figure S8D). Based on these observations, we hypothesize that this histological abnormality appears to be related to a defect in epithelial morphogenesis.

To address this possibility in greater detail, we analyzed the localization of AB polarity markers. Activated aPKC (phospho-aPKC) has been implicated in the establishment of AB polarity in mammalian cells [23]. We observed proper localization of aPKC to the apical region of control small intestine epithelium. In contrast, specific sub-cellular localization of aPKC disappeared in a clump of epithelial cells in *Sfrp1−/− Sfrp2−/− Sfrp5+/−* small intestine (Figure 5B–G). Antibody staining against ß1-integrin revealed a round cell shape in the clump of epithelium (Figure 5D, E; Video S1, S2). In addition, E-cadherin was concentrated at the apicolateral cytoplasmic membrane in control epithelium (Figure 5F). However, it was widely distributed in the cytoplasmic membrane of the epithelial cell clump (Figure 5G, asterisk; Video S3, S4). These observations indicate the involvement of Type 1 Sfrps in the regulation of AB polarity in the small intestine epithelium.

**Figure 6:**
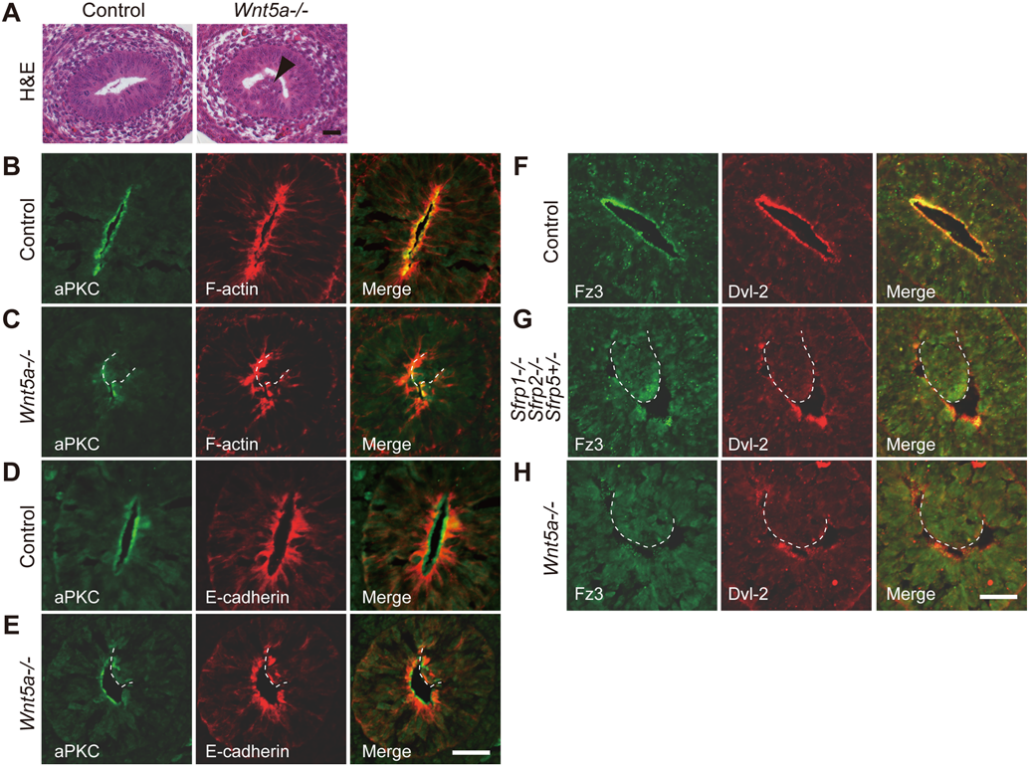
Defective epithelial AB polarity in *Wnt5a* *−* */* *−* small intestine. (A) A clump of epithelial cells in the *Wnt5a−/−* small intestine at E13.5 as visualized by H&E staining (arrowhead). (B–E) AB cell polarity is lost in the epithelial clump of the *Wnt5a-*inactivated small intestine, as suggested by aPKC (B–E), F-actin (B, C) and E-cadherin (D, E) sub-cellular localization. Scale bar: 25 µm. (F–H) Sub-cellular distribution of the Fz/PCP pathway components in the epithelium of *Sfrp1−/− Sfrp2−/− Sfrp5+/−* and *Wnt5a−/−* small intestine at E13.5. Fz3 and Dvl2 localize to the apical region of control small intestine epithelium (F). Apical localization disappears in the epithelial cell clump of *Sfrp1−/− Sfrp2−/− Sfrp5+/−* small intestine (G). *Wnt5a* inactivation affects sub-cellular distribution of Fz3 and Dvl2 in small intestine epithelium (H). Scale bar: 25 µm.

Since a relationship between AB polarity and the PCP pathway was suggested previously [30],[36], we assessed sub-cellular distributions of Fz3 and Dvl-2 in the small intestine. In control epithelium derived from small intestine corresponding to the jejunum and the ileum at E13.5, Fz3 occupied the apical region and co-localized with Dvl-2 (Figure 6F). However, Fz3 and Dvl-2 were not concentrated in the apical region of the *Sfrps*-deficient small intestine epithelium, especially in the clump of epithelial cells (Figure 6G). No difference in sub-cellular distributions of Fz3 and Dvl-2 was detected in other regions of the *Sfrp1−/− Sfrp2−/− Sfrp5+/−* gut tube relative to that of the control gut tube. Thus, Type 1 Sfrps affect AB cell polarity in conjunction with the regulation of the sub-cellular distribution of core Fz/PCP factors in the small intestine epithelium.

### AB Polarity in *Wnt5*a*−*/*−* Small Intestine Epithelium

TOPGAL reporter activity [24] indicated that up-regulation did not occur in the canonical pathway within the *Sfrp1−/− Sfrp2−/− Sfrp5+/−* small intestine epithelium (Figure S9A, B). Since similar defects in terms of oriented cell division were observed in the stomachs of *Sfrp1−/− Sfrp2−/− Sfrp5+/−* and Wnt5a inactivated embryos, we examined AB polarity in the small intestine of *Wnt5a−/−* embryos at E13.5. The epithelial structure was disrupted by epithelial cell clumps in the region corresponding to the jejunum and the ileum of *Wnt5a−*/*−* embryos (Figure 6A). In addition, the apical distribution of aPKC was disturbed in *Wnt5*a*−*/*−* small intestine epithelium (Figure 6B–E), suggesting a defect in AB polarity. Moreover, this epithelial abnormality was associated with defective Fz3 and Dvl-2 sub-cellular distributions in the *Wnt5a−*/*−* small intestine (Figure 6H). The gut phenotypes observed in *Sfrp1−/− Sfrp2−/− Sfrp5+/−* embryos appeared to be more severe than those in *Wnt5a−/−* embryos. However, *Wnt5a−/−* embryos displayed significant phenotypic similarities to *Sfrps*-deficient embryos.

### Sfrps Modulate Wn5a Signaling

Although the morphological abnormalities detected in the gut of *Wnt5a−/−* embryos resembled those in *Sfrps*-deficient gut, this observation did not necessarily equate to a similarity in signaling regulation. In fact, the loss or gain of Wnt5a function results in dys-regulated convergent extension (CE) movements in vertebrates [37],[38]. Additionally, previous reports imply that Sfrp2 antagonizes Wnt5a signaling [39].

**Figure 7:**
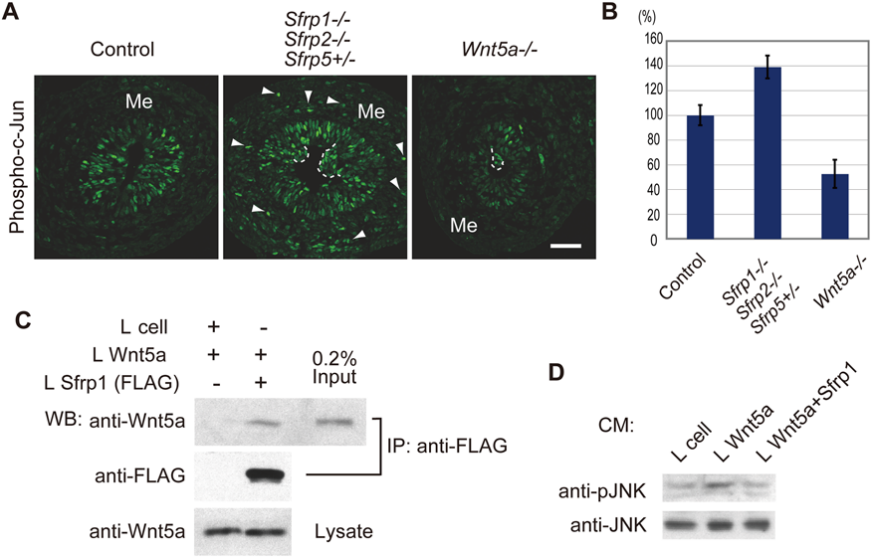
Sfrps inhibit Wnt5a signaling. (A, B) The level of phospho-c-Jun is elevated in *Sfrp1−/− Sfrp2−/− Sfrp5+/−* small intestine epithelium as well as in mesenchyme at E13.5. These levels are reduced by *Wnt5a* inactivation. Scale bar: 50 µm. The activation levels in the epithelium are measured as the intensity of antibody staining (B). (C) Sfrp1 interacts with Wnt5a. Sfrp1-FLAG (IP, lower) co-precipitates Wnt5a (IP, upper) from the cell lysate containing Sfrp1-FLAG and Wnt5a (Lysate) in a co-culture immunoprecipitation assay involving anti-FLAG antibody. (D) Sfrp1 inhibits Wnt5a signaling. The level of phospho-JNK indicates pathway activation induced by Wnt5a in conditioned medium (CM).

To establish a molecular relationship between Sfrps function and Wnt5a signaling, the signaling activity of the Wnt5a pathway in the gut was assayed in terms of phospho c-Jun levels. It is well established that Wnt5a activates c-Jun N-terminal kinase (JNK). In turn, JNK phosphorylates c-Jun [40],[41]. Currently, phospho c-Jun is the only available marker in the pathway detectable with antibody staining (Figure 7A). Phospho c-Jun-positive cells were observed in small intestine epithelium at E13.5; however, it was scarcely detected in fore-stomach epithelium. In *Sfrps*-deficient small intestine, phospho c-Jun levels were elevated significantly in the epithelium in comparison with the control small intestine epithelium (Figure 7A, B). Moreover, phospho c-Jun-positive cells were frequently observed in the mesenchyme of *Sfrp1−/− Sfrp2−/− Sfrp5+/−* embryos (Figure 7A). In contrast, phospho-c-Jun levels were decreased in *Wnt5a−/−* epithelium (Figure 7A, B). Immunofluorescence staining was repeated three times, followed by statistical analysis of the staining intensity. The results were statistically significant (i.e., Control, 100±8.16%; Sfrps-deficient, 139±9.24%; Wnt5a*−*/*−*, 52.5±11.4%; P<0.01) upon comparison between control and Sfrps-deficient or Wnt5a*−*/*−* small intestines (Figure 7 B). These observations suggest that Type 1 Sfrps may inhibit Wnt5a signaling. Therefore, we tested this possibility. In a co-culture immunoprecipitation assay, Wnt5a co-precipitated with Sfrp1 from the lysate containing Sfrp1 and Wnt5a; this observation indicated that Sfrp1 interacts with Wnt5a (Figure 7C). We also examined the effect of Sfrp on JNK activation induced by Wnt5a. In conditioned medium, Wnt5a elevated the levels of phospho-JNK, an active form, in HEK293T cells. However, Sfrp1, in the presence of Wnt5a, reduced the levels of active JNK (Figure 7D). Thus, Type 1 Sfrps are capable of inhibiting Wnt5a signaling.

## Discussion

### Oriented Cell Division in Epithelium Is Associated with Fore-Stomach Morphogenesis

In Zebrafish, oriented cell division is a driving force of a-p axis elongation during gastrulation and neural tube morphogenesis during neurulation [42]–[44]. Moreover, in *Drosophila,* oriented cell division in the imaginal disc affects adult organ shape [45]. In both processes, oriented cell division is disrupted by a defect in the Fz/PCP pathway [42],[43],[45]. Thus, the Fz/PCP pathway regulates cell division orientation with respect to tissue elongation during embryonic development. However, this involvement was unknown in mammals.

We observed divergence of cell division orientation in the greater curvature of the fore-stomach in *Sfrps*-deficient and *Wnt5a−/−* embryos. Both *Wnt5a* and Type1 *Sfrps* genetically interact with *Stbm/Vangl2* [9],[31]. Moreover, oriented cell division was disrupted in the fore-stomach epithelium of *Lp/Lp* embryos. Thus, these observations are indicative of Fz/PCP pathway regulation of epithelial oriented cell division in the fore-stomach. Significantly, the components of the Fz/PCP pathway, i.e., Fz3, Dvl2 and Vangl2, are expressed in the epithelium [27]; in contrast, co-expression is not evident in the mesenchyme. Furthermore, sub-cellular distribution of Fz3 and Dvl-2 was affected in *Sfrps*-deficient fore-stomach epithelium. Therefore, the Fz/PCP pathway components in the epithelium appear to be involved in the regulation of oriented cell division.

Following the initiation of organ bud formation at around E10.5, the stomach is dramatically enlarged over several days. Previous data of Nyeng et al. [26] suggest that the epithelium of the fore-stomach initiates terminal differentiation at E15.5. Hence, most of the cells in fore-stomach epithelium at E12.5 and E13.5 could be immature cells continuing cell division during these stages. When we observed cell division in fixed samples, approximately 3% of epithelial cells demonstrated division in the greater curvature of the fore-stomach at E12.5. However, cell proliferation occurs rapidly in the epithelium; additionally, an increment of epithelial cell number generates the largest number of dividing cells in the entire fore-stomach epithelium during organ development. Therefore, cell division orientation could be one of essential components contributing to fore-stomach morphogenesis.

The phenotype described by fore-stomach shortening and mitotic orientation defect in *Sfrp1−/− Sfrp2−/− Sfrp5+/−* stomachs might be most severe in all mutants; additionally, the phenotype in *Lp/Lp* stomachs might be relatively milder in comparison to that found in other mutants. Although the difference between the mitotic orientation defects of the mutants was statistically insignificant, a weak tendency in the severity of mitotic orientation defect, which could be correlated with the severity of fore-stomach shortening defects, may occur. The weak tendency might be due to our observation of cell division involving an instantaneous event of an individual cell, whereas the morphological features of the fore-stomach shortening defect was a result of accumulated cellular events.

A defect in the cell rearrangement process (e.g., increased radial intercalation) [46] might be a possible element in the induction of fore-stomach shortening. However, epithelial cell division along the AB axis occurred at low frequencies and the mono-cell layer was maintained in *Sfrps*-deficient fore-stomach epithelium at E12.5. Therefore, oriented cell division and allocation of the divided cells along the cell division axis could contribute to organ lengthening as one of the earlier events in fore-stomach morphogenesis. The fore-stomach is the most remarkable structure in the developing gut tube, which is generated during mid-gestation. Most internal organs are generated from an epithelial tubule structure, with the tube altering its shape depending on the function of the organ. It is possible that oriented cell division is a common mechanism that is essential for tubular morphogenesis of the internal organs.

Our results implicate the Fz/PCP pathway, in association with Sfrp regulation of Wnt5a, in the regulation of oriented cell division. The reason that mutations in opposing regulatory components lead to similar defects in oriented cell division may be due to the loss of planer cell polarity in both defects. Animals with mutations in opposing Fz/PCP pathway regulatory components frequently exhibit similar phenotypes [47].

The current in vitro data and data representing the response of downstream effectors of Wnt5a signaling suggested that Sfrp inhibits Wnt5a signaling. Genetic analysis was also conducted via the generation of *Wnt5a*, *Sfrp1* and *Sfrp2* compound mutant embryos. A survey of the morphology and internal organs of these embryos was suggestive of no rescue and no enhanced phenotype in *Wnt5a+/− Sfrp1−/− Sfrp2−/−* embryos in comparison with *Sfrp1−/− Sfrp2−/−* embryos at E10.5, E12.5 and E13.5. Thus, the genetic analysis was not beneficial in terms of evaluation of an interaction between Sfrps function and Wnt5a signaling. This observation is likely attributable to the insufficient capacity of the heterozygous mutation to reduce Wnt protein expression under an effective dosage.

The Wnt5a transcript is highly expressed in fore-stomach mesenchyme; in contrast, the expression is weaker in hind-stomach mesenchyme at E13.5. Based on transcriptional expression patterns, an active Wnt5a protein gradient may be generated in conjunction with Sfrps along the cephalocaudal axis in the stomach. Further investigation is necessary in order to understand the mechanism by which the protein gradient is involved in epithelial oriented cell division through the Fz/PCP pathway.

### Wnt Regulation of AB Polarity in Small Intestine Epithelium

Previous studies have identified the role of various molecules including the conserved PAR-aPKC complex in the regulation of epithelial AB polarity [23]. Additionally, the involvement of PCP pathway components in AB polarity of epithelial cells has been suggested [36],[48]. However, little is known regarding intracellular signaling regulation. The intercellular signaling regulation could coordinate AB polarity in developing organs. We observed a similar AB polarity defect in both Sfrps-deficient and Wnt5a inactivated small intestines. In contrast, we could not identify clear evidence suggesting abnormality of AB polarity in the fore-stomach; AB polarity was not well established even in wild-type fore-stomach epithelium throughout the assessment of sub-cellular distribution of protein markers.

Although Sfrps are capable of inhibiting Wnt5a signaling, it is possible that mutations in opposite regulatory components result in similar defects as both defects lead to a loss of AB polarity. Previously published data indicate that Wnt5a activates JNK [40],[41], which is an essential effecter of CE movement [49]. JNK phoshorylates paxillin, a component of the focal adhesion complex [50]. In addition, Wnt5a is able to activate focal adhesion kinase (FAK) [39]. Paxillin and FAK are known to play a role in cell migration [39],[50]; thus, the possibility exists that Wnt5a promotes cell migration. However, FAK and paxillin are also required for the maintenance of adherence junctions via N-cadherin-based cell-cell adhesion. Regarding this function, FAK and paxillin are involved in a mechanism of the down-regulation of the activity of the small GTP-binding protein Rac1 [51]. In contrast, Wnt5a signaling is also involved in the up-regulation of Rac1 [39]. Interestingly, the constitutive active and dominant negative forms of Rac1 lead to a phenotype similar to that observed upon the loss of E-cadherin at sites of cell-cell contact [52],[53]. Furthermore, E-cadherin provides a clue with respect to the development of AB polarity leading to the recruitment of the PAR-aPKC complex to immature adherence junctions [23]. In this respect, appropriate levels of Wnt5a signaling activity may be essential for the modulation of AB polarity. Hence, we propose that Sfrps and Wnt5a are putative components of intracellular signaling regulation in order to coordinate AB polarity in the developing small intestine.

Control of AB polarity via Wnt signaling has been suggested. In *Xenopus* and *Drosophila*, Dvl is necessary for basolateral membrane localization of Lgl (Lethal giant larvae), which encodes a protein with multiple WD-40 motifs that regulates AB polarity [48]. Dvl interacts with Lgl. Moreover, Fz8, but not Fz3 and Fz7, regulate Lgl sub-cellular localization [48], which suggests that the basolateral localization of Fz8, Dvl and Lgl is required for the establishment of AB polarity [48]. In contrast, we demonstrated that Dvl-2 is concentrated at the apical surface of gut epithelium and that this apical localization overlaps with Fz3 localization. Thus, these observations suggest that AB polarity regulation in gut epithelium is distinct from that previously described in *Xenopus* and *Drosophila.*

In summary, our results indicate a link between Sfrps function and Wnt5a signaling in the regulation of epithelial cell polarity including oriented cell division and AB cell polarity in developing gut. Sfrps are known as tumor suppressor genes, which are epigenetically silenced in many types of cancer, especially in colorectal cancer [7]. In contrast, up-regulation and down-regulation of Wnt5a are observed in gastric and colon cancer, as well as in cancer progression [39],[54]. Sfrps-regulation of Wnt5a signaling may provide novel insight into the progression and aggressiveness of GI tract cancer.

## Materials and Methods

### Mice

*Sfrp1−/− Sfrp2+/−* and *Sfrp1−/− Sfrp2+/− Sfrp5−/−* mice were maintained in a 129 and C57BL/6 mixed genetic background [9],[12]. *Sfrp1−/− Sfrp2−/− Sfrp5+/−* embryos were derived from crosses between *Sfrp1−/− Sfrp2+/−* and *Sfrp1−/− Sfrp2+/− Sfrp5−/−* mice. *Lp* (*LPT/LeJ*) mice and *Wnt5a* heterozygous mutant (*Wnt5^atm1Amc^/J*) mice were obtained from the Jackson Laboratory [31],[55]. Crosses were utilized to introduce the TOPGAL reporter [24] into Sfrps-deficient and *Wnt5a+/−* mice. The activity of the TOPGAL reporter was visualized via standard LacZ staining involving a short reaction time of 30 minutes to compare activation levels.

### *In Situ* Hybridization

An *Sfrp1* cDNA fragment obtained from cDNA subtraction screening was used as a probe for whole mount *in situ* hybridization [56]. *Sfrp2*, *Sfrp5* and *Barx1* cDNA clones were obtained as I.M.A.G.E. clones; *Cdx2*, *Hoxa4, Hoxd13*, *Islet1*, *Nkx6.3* and *Pdx1* probes were generated from FANTOM cDNA clones [57].

### Immunofluorescence Staining and Immunoprecipitation

The GI tract was isolated from control, *Sfrp1−/− Sfrp2−/−, Sfrp1−/− Sfrp2−/− Sfrp5+/−*, *Lp/Lp* and *Wnt5a*−/− embryos in phosphate buffered saline (PBS) containing 10% fetal calf serum. Whole mount immunofluorescence staining of stomach epithelium was performed as described previously [12] employing anti-acetylated α-tubulin antibody (Sigma, mouse monoclonal clone 6-11B-1) and anti-ß1-integrin antibody (Chemicon, rat monoclonal MAB1997). Chromosomes were visualized by DAPI staining. Images were captured on a BioRad Radiance 2100 Laser Scanning Confocal Microscope System equipped with a Zeiss Axiovert and processed using Adobe Photoshop. Immunofluorescence staining of sectioned tissue was conducted utilizing the following primary antibodies: anti-ß-galactosidase antibody (from Rabbit, Cappel), anti-E-cadherin antibody (Sigma, mouse monoclonal anti-Uvomorulin clone DECMA-1), anti-phospho-aPKC antibody (from rabbit, Cell Signaling), anti-Fz3 antibody (from rabbit, MBL), anti-Dvl-2 antibody (from goat, Santa Cruz, N-19), anti-FoxA2 antibody [58] (from Rabbit) and anti-Sox2 antibody (from goat, Santa Cruz, Y-17), anti-phospho-c-Jun (from Rabbit, Cell Signaling) and anti-BrdU (mouse monoclonal, BD). F-actin was visualized with Rhodamine-conjugated phalloidin. Images, which were captured on a BioRad Radiance 2100 Laser Scanning Confocal Microscope System equipped with a Zeiss Axiovert, were processed using Adobe Photoshop. Images of anti-phospho-c-Jun staining were analyzed by imaging software MultiGauge (Fujifilm) to calculate averages of signaling intensity per area in the epithelium. With respect to this calculation process, staining background intensity in epithelium and mesenchyme was measured, followed by subtraction from the specific signal intensity. For the BrdU incorporation assay, BrdU/PBS solution was injected into a pregnant mouse intraperitoneally (100 µg/g body weight) 1 h before embryo collection.

The immunoprecipitation assay was performed by co-culture immunoprecipitation of L cells expressing Wnt5a (L Wnt-5A, ATCC) and L cells expressing Sfrp1-FLAG (c-terminal tagged with FLAG, L Sfrp1) (1∶1 ratio). Sfrp1-FLAG was precipitated with anti-FLAG M2 affinity gel (Sigma) according to the manufacturer's protocol. Goat anti-Wnt5a antibody (R&D Systems, Inc.) was used to detect Wnt5a protein. JNK activity was evaluated based on the levels of phospho-JNK determined by an anti-phospho-JNK antibody (Cell Signaling). Total JNK was detected by anti-JNK antibody (Cell Signaling). Lysates for Western blotting were derived from HEK293T cells incubated for 3 h in L cell conditioned medium. L Sfrp1 was established as a stable transformant cell line. Conditioned media from L cell, L Wnt5a and L Sfrp1 were obtained following a 4-day incubation of the culture medium.

### Epithelial Cell Counting

Histological sections (5 µm) stained with Hematoxylin and Eosin (H&E) were prepared from Bouin-fixed and paraffin-embedded specimens. Cell number per 2000 µm^2^ in fore-stomach epithelium was calculated by counting the nuclei in a 400-µm wide area of a single section. Total cell number in an epithelial cell suspension was determined with a hemocytometer. Cell suspensions were prepared from stomach epithelium separated from mesenchyme following incubation of the sample in PBS containing 0.5 unit dispase and 1.25% pancreatin at room temperature for 30 minutes. Statistical significance, which was evaluated using Welch's t-test, was defined as P<0.05. Error bars indicate SD.

### Ethics Statement

All animals were handled in strict accordance with good animal practice as defined by the relevant national and/or local animal welfare bodies, and all animal works were approved by the appropriate committee.

## Supporting Information

Figure S1 *Sfrp1*, *Sfrp2* and *Sfrp5* expression in the developing gut. (A–I) *Sfrp1* (A, D, G), *Sfrp2* (B, E, H) and *Sfrp5* (C, F, I) are expressed in the gut tube at E10.5 (A–C) and E12.5 (D–I). Ce, cecum; Co, colon; Es, esophagus; Hg, hindgut; Mg, midgut; St, stomach. G, H and I are opposite sides of the gut shown in D, E and F, respectively. The arrowheads indicate *Sfrp2* expression in the colon (H). Scale bar: 500 µm. (J–M) *Sfrp1*, *Sfrp2* and *Sfrp5* expression in gut epithelium and mesenchyme. The sections were generated from samples following *in situ* hybridization. *Sfrp1* expression is apparent in the mesenchyme of the gut tube (J). *Sfrp2* expression is observed in a portion of the mesenchyme in the esophagus (K) and in the fore-stomach and colon epithelium (L). *Sfrp5* is expressed in the epithelium from the duodenum to the jejunum (M). Ep, epithelium; Me, mesenchyme. Scale bar: 50 µm. (N) *Sfrp1*, *Sfrp2* and *Sfrp5* expression in the developing gut tube.
(3.41 MB TIF)

Figure S2 *Sfrps*-deficiency results in gut tube malformation. (A–C) Gross morphology of control (A), *Sfrp1−/− Sfrp2−/−* (B) and *Sfrp1−/− Sfrp2−/− Sfrp5+/−* (C) embryos at E13.5. The arrowheads indicate edema. Scale bar: 1 mm. (D–F) The length/size of the stomach and small intestine is reduced in *Sfrp1−/− Sfrp2−/−* (E) and *Sfrp1−/− Sfrp2−/− Sfrp5+/−* (F) embryos in comparison with controls (D). Ce, cecum; Co, colon; Es, esophagus; St, stomach. Scale bar: 500 µm.
(1.67 MB TIF)

Figure S3 Epithelial differentiation in the glandular and non-glandular stomach of control and *Sfrp1−/− Sfrp2−/− Sfrp5+/−* embryos at E16.5. (A, B) Histological sections of control (A) and *Sfrp1−/− Sfrp2−/− Sfrp5+/−* (B) stomachs at E16.5. The arrowhead denotes the boundary of the glandular and non-glandular stomach. Scale bar: 500 µm. (C–F) Characteristic epithelial structure and cell types are observed in *Sfrp1−/− Sfrp2−/− Sfrp5+/−* (D, F) and control stomachs (C, E). The arrow identifies a portion of the mucosa in the non-glandular stomach. The positions of C, D, E and F are indicated in A and B. Scale bar: 100 µm.
(4.89 MB TIF)

Figure S4 Shortening along the cephalocaudal axis and lateral expansion of *Sfrp1−/− Sfrp2−/− Sfrp5+/−* fore-stomach at E12.5. (A) Ventral (upper) and posterior (lower) view of control and *Sfrp1−/− Sfrp2−/− Sfrp5+/−* stomachs at E12.5. Scale bar: 500 µm. Fu, fundus; Du, duodenum. (B, C) The length of the greater curvature epithelium was shortened in *Sfrp1−/− Sfrp2−/− Sfrp5+/−* fore-stomach (between arrow and arrowhead in A) in comparison with control fore-stomach (B). In contrast, the width at the junction of the fundus and the body was increased in *Sfrp1−/− Sfrp2−/− Sfrp5+/−* fore-stomach (C). (D) The mono-cell layer structure of the greater curvature of control and *Sfrps*-deficient fore-stomachs at E12.5. Ep, epithelium. The arrowhead indicates the basement membrane. Scale bar: 50 µm. (E) Cell number per area (2000 µm^2^) of control (37.6±4.59 cells, n = 3) and *Sfrp1−/− Sfrp2−/− Sfrp5+/−* (48.5±7.43 cells, n = 3) fore-stomach epithelium. (F) Frequency of multi-nuclei along the AB axis in the greater curvature epithelium of control and Sfrps-deficient fore-stomachs (4.37±2.01% of 343 control epithelial cells, n = 3; 4.11±1.30% of 438 *Sfrp1−/− Sfrp2−/− Sfrp5+/−* epithelial cells, n = 3).
(2.28 MB TIF)

Figure S5 *Sfrp1−/− Sfrp2−/− Sfrp5+/−* fore-stomach epithelium at E13.5. (A, B) The greater curvature epithelium of control (A) and *Sfrp1−/− Sfrp2−/− Sfrp5+/−* (B) fore-stomachs. Ep, epithelium. The arrowhead indicates the basement membrane. Scale bar: 50 µm. (C, D) Total cell number in the epithelium is unaltered in *Sfrp1−/− Sfrp2−/− Sfrp5+/−* fore-stomach (C; 3.21±0.32×10^4^ cells, n = 3) as well as in *Sfrp1−/− Sfrp2−/− Sfrp5+/−* hind-stomach (D; 3.59±0.45×10^4^ cells, n = 3) in comparison to the control fore- (3.36±0.24×10^4^ cells, n = 3) and hind-stomach (3.75±0.47×10^4^ cells, n = 3). (E, F) Epithelial cell number per area (2000 µm^2^) increased approximately 27% in the greater curvature of *Sfrp1−/− Sfrp2−/− Sfrp5+/−* fore-stomach (52.9±1.92 cells) versus that of control (E; 41.6±1.73 cells, n = 4); however, no difference was observed in the hind-stomachs derived from control (83.3±4.8 cells) and *Sfrp1−/− Sfrp2−/− Sfrp5+/−* (82.5±5.5 cells) embryos (F; n = 4).
(1.53 MB TIF)

Figure S6 The Wnt/β-catenin pathway in Sfrps-deficient stomach epithelium. (A, B) TOPGAL activity visualized by anti-β-galactosidase antibody staining indicates higher canonical Wnt/β-catenin signaling activity in control (A) and *Sfrp1−/− Sfrp2−/− Sfrp5+/−* (B) fore-stomachs at E13.5. Scale bar: 200 µm. (C, D) Reporter activity indicates slightly enhanced canonical Wnt/β-catenin signaling in *Sfrp1−/− Sfrp2−/− Sfrp5+/−* hind-stomach epithelium (D) in comparison with the control (C). Scale bar: 50 µm. Note that β-galactosidase derived from the TOPGAL reporter exhibits cytoplasmic localization in the epithelium (Ep), whereas β-galactosidase derived from the *Sfrp1* knock-in locus displays nuclear localization in the mesenchyme (Me). The arrowhead identifies a boundary between fore- and hind-stomach epithelium as determined by epithelial morphology. (E–J) Protein distribution of FoxA2 and Sox2 is not significantly altered in the junction between the fore- and hind-stomach in *Sfrp1−/− Sfrp2−/− Sfrp5+/−* embryos in comparison to control embryos at E13.5. Higher FoxA2 expression is found in fore-stomach epithelium; in contrast, the expression weakens at the junction of the fore- and hind-stomach epithelium (arrow). Sox2 expression in fore-stomach epithelium is gradually reduced in the hind-stomach epithelium (bracket). Scale bar: 50 µm.
(2.83 MB TIF)

Figure S7 Regionalization of the intestine in *Sfrp1−/− Sfrp2−/−* and *Sfrp1−/− Sfrp2−/− Sfrp5+/−* embryos at E13.5. (A–E) The intestines of *Sfrp1−/− Sfrp2−/−* and *Sfrp1−/− Sfrp2−/− Sfrp5+/−* embryos are regionalized along the cephalocaudal axis, as suggested by the expressions of *Cdx2* (A), *Sfrp5* (B), *Hoxa4* (C), *Wnt5a* (D) and *Hoxd13* (E). Ce, cecum; Co, colon; St, stomach. Scale bar: 500 µm. (F) The length of the small intestine was reduced in *Sfrp1−/− Sfrp2−/−* embryos (5.5±0.39 mm; n = 9; P<0.0001) relative to control small intestine at E13.5 (10.1±1.32 mm; n = 9). The reduction in length was enhanced upon introduction of an *Sfrp5* mutant allele (3.7±0.37 mm; n = 9; P<0.0001). (G) The length of the rostral large intestine coinciding with the *Hoxd13*-negative region is shortened in *Sfrp1−/− Sfrp2−/−* (1.30±0.076 mm; n = 8; P<0.0001) and *Sfrp1−/− Sfrp2−/− Sfrp5+/−* (1.31±0.11 mm; n = 8; P<0.0001) embryos in comparison with controls (2.73±0.18 mm; n = 9) at E13.5.
(3.81 MB TIF)

Figure S8 BrdU incorporation assay in *Sfrps*-deficient small intestine. (A–C) Cell proliferation ratios increased in neither the epithelial cell clump (A, B) nor the entire epithelium (C) in the *Sfrp1−/− Sfrp2−/− Sfrp5+/−* small intestine in comparison with control small intestine at E13.5. Scale bar: 50 µm. (D) Cell density was not significantly increased in the epithelium of *Sfrp1−/− Sfrp2−/− Sfrp5+/−* small intestine (11.3±0.32 cells/1000 µm^2^, n = 3) in comparison with control epithelium (10.1±0.87 cells/1000 µm^2^, n = 3) at E13.5.
(1.78 MB TIF)

Figure S9 The Wnt/ß-catenin pathway is not enhanced in *Sfrp1−/− Sfrp2−/− Sfrp5+/−* small intestine. (A, B) TOPGAL activity visualized by anti-β-galactosidase antibody staining indicates that Wnt/β-catenin signaling activity is not enhanced in *Sfrp1−/− Sfrp2−/− Sfrp5+/−* small intestine epithelium at E13.5. Scale bar: 50 µm.
(1.00 MB TIF)

Video S1 Epithelial cell morphology in the control small intestine revealed by ß1-integrin distribution pattern.
(0.35 MB MOV)

Video S2 Epithelial cell morphology in the Sfrps-deficient small intestine revealed by ß1-integrin distribution pattern.
(0.35 MB MOV)

Video S3 E-cadherin distribution pattern in control small intestine epithelium.
(0.34 MB MOV)

Video S4 E-cadherin distribution pattern in Sfrps-deficient small intestine epithelium.
(0.36 MB MOV)
