# Supplementary figures and images for: Sfrp Controls Apicobasal Polarity and Oriented Cell Division in Developing Gut Epithelium
Source: PLoS Genet. 2009 Mar 20;5(3):e1000427. doi: 10.1371/journal.pgen.1000427 (PMC2649445; doi:10.1371/journal.pgen.1000427)

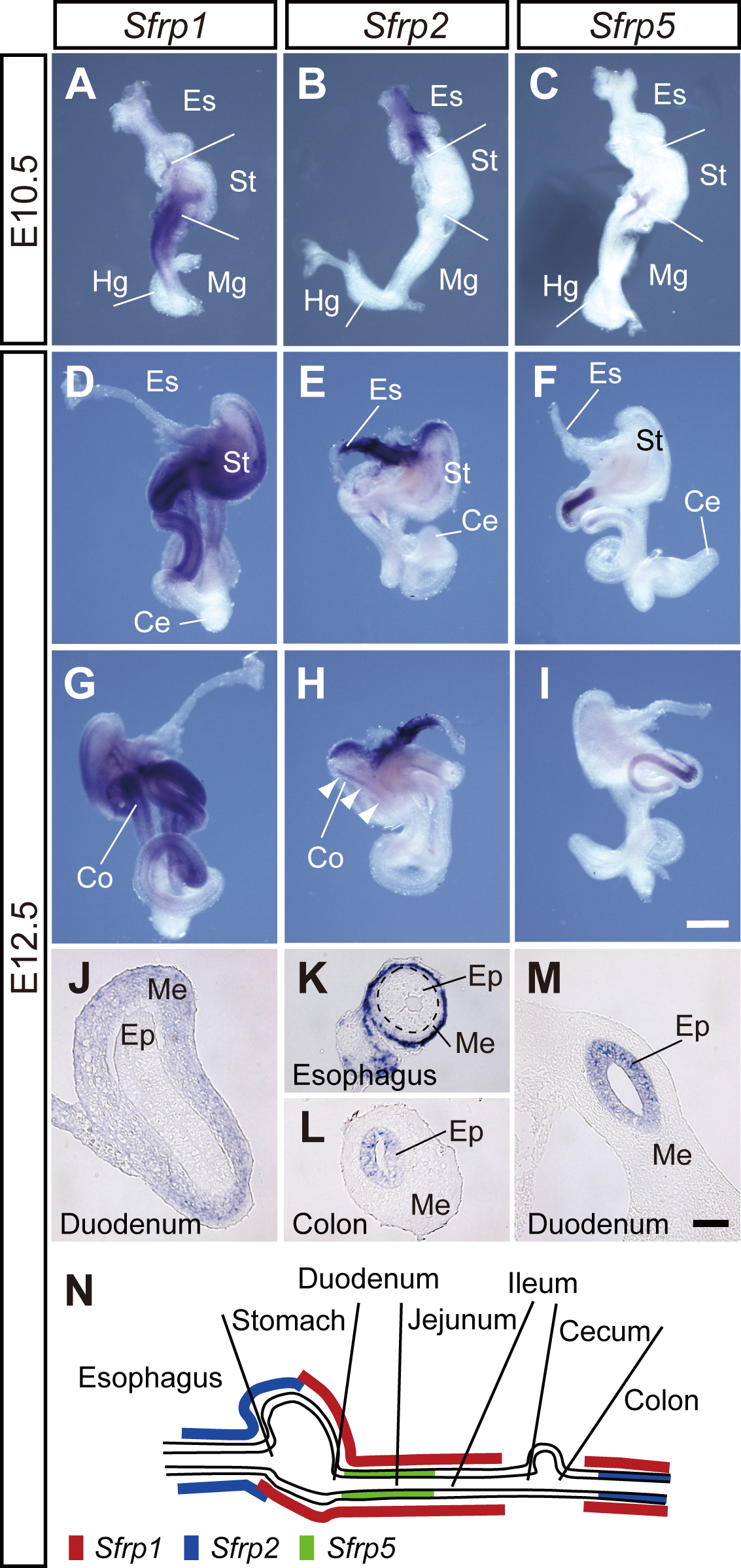

Supplement: Figure S1 — Sfrp1, Sfrp2 and Sfrp5 expression in the developing gut. (A–I) Sfrp1 (A, D, G), Sfrp2 (B, E, H) and Sfrp5 (C, F, I) are expressed in the gut tube at E10.5 (A–C) and E12.5 (D–I). Ce, cecum; Co, colon; Es, esophagus; Hg, hindgut; Mg, midgut; St, stomach. G, H and I are opposite sides of the gut shown in D, E and F, respectively. The arrowheads indicate Sfrp2 expression in the colon (H). Scale bar: 500 µm. (J–M) Sfrp1, Sfrp2 and Sfrp5 expression in gut epithelium and mesenchyme. The sections were generated from samples following in situ hybridization. Sfrp1 expression is apparent in the mesenchyme of the gut tube (J). Sfrp2 expression is observed in a portion of the mesenchyme in the esophagus (K) and in the fore-stomach and colon epithelium (L). Sfrp5 is expressed in the epithelium from the duodenum to the jejunum (M). Ep, epithelium; Me, mesenchyme. Scale bar: 50 µm. (N) Sfrp1, Sfrp2 and Sfrp5 expression in the developing gut tube. (3.41 MB TIF) [file pgen.1000427.s001.tif]

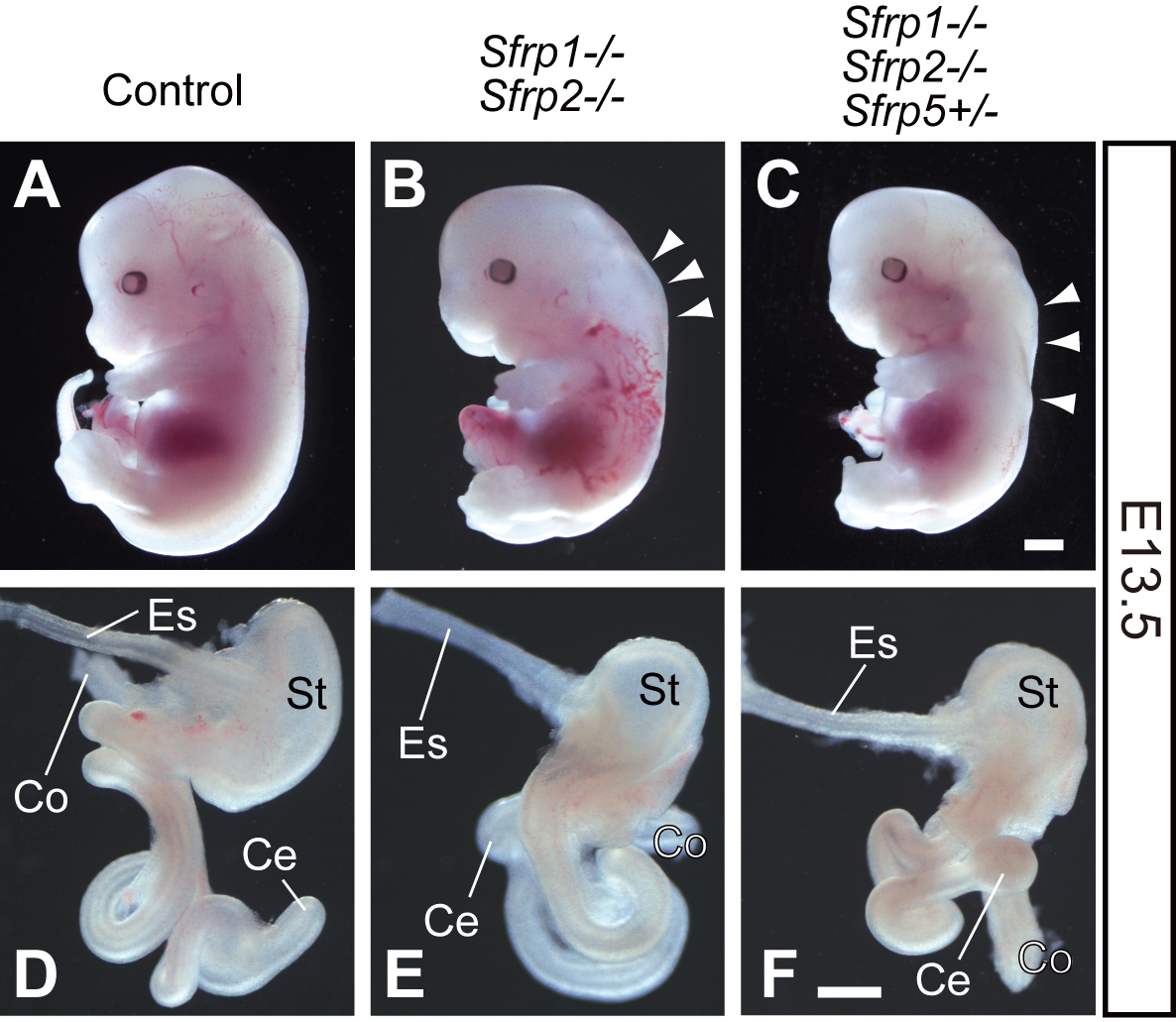

Supplement: Figure S2 — Sfrps-deficiency results in gut tube malformation. (A–C) Gross morphology of control (A), Sfrp1−/− Sfrp2−/− (B) and Sfrp1−/− Sfrp2−/− Sfrp5+/− (C) embryos at E13.5. The arrowheads indicate edema. Scale bar: 1 mm. (D–F) The length/size of the stomach and small intestine is reduced in Sfrp1−/− Sfrp2−/− (E) and Sfrp1−/− Sfrp2−/− Sfrp5+/− (F) embryos in comparison with controls (D). Ce, cecum; Co, colon; Es, esophagus; St, stomach. Scale bar: 500 µm. (1.67 MB TIF) [file pgen.1000427.s002.tif]

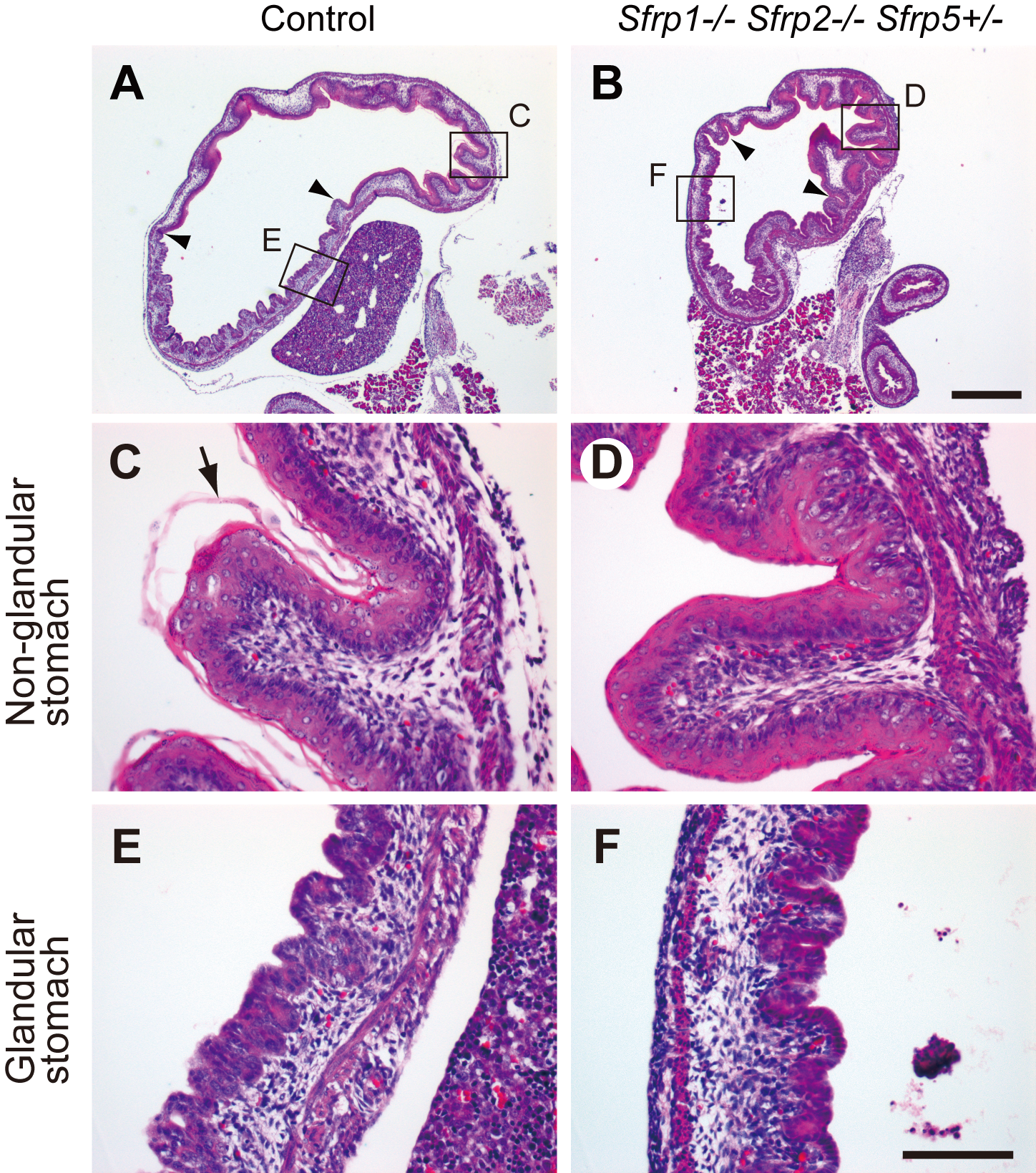

Supplement: Figure S3 — Epithelial differentiation in the glandular and non-glandular stomach of control and Sfrp1−/− Sfrp2−/− Sfrp5+/− embryos at E16.5. (A, B) Histological sections of control (A) and Sfrp1−/− Sfrp2−/− Sfrp5+/− (B) stomachs at E16.5. The arrowhead denotes the boundary of the glandular and non-glandular stomach. Scale bar: 500 µm. (C–F) Characteristic epithelial structure and cell types are observed in Sfrp1−/− Sfrp2−/− Sfrp5+/− (D, F) and control stomachs (C, E). The arrow identifies a portion of the mucosa in the non-glandular stomach. The positions of C, D, E and F are indicated in A and B. Scale bar: 100 µm. (4.89 MB TIF) [file pgen.1000427.s003.tif]

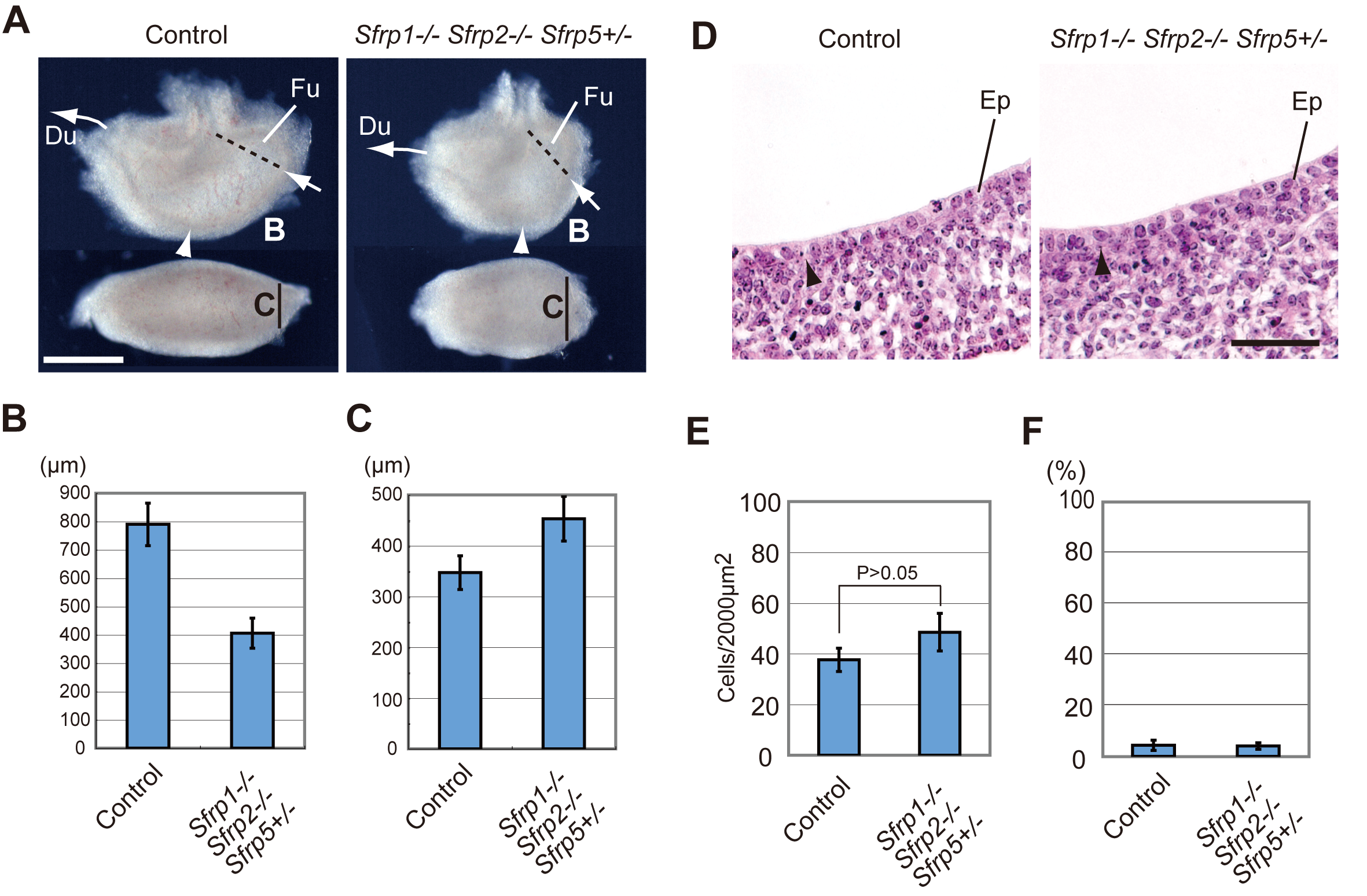

Supplement: Figure S4 — Shortening along the cephalocaudal axis and lateral expansion of Sfrp1−/− Sfrp2−/− Sfrp5+/− fore-stomach at E12.5. (A) Ventral (upper) and posterior (lower) view of control and Sfrp1−/− Sfrp2−/− Sfrp5+/− stomachs at E12.5. Scale bar: 500 µm. Fu, fundus; Du, duodenum. (B, C) The length of the greater curvature epithelium was shortened in Sfrp1−/− Sfrp2−/− Sfrp5+/− fore-stomach (between arrow and arrowhead in A) in comparison with control fore-stomach (B). In contrast, the width at the junction of the fundus and the body was increased in Sfrp1−/− Sfrp2−/− Sfrp5+/− fore-stomach (C). (D) The mono-cell layer structure of the greater curvature of control and Sfrps-deficient fore-stomachs at E12.5. Ep, epithelium. The arrowhead indicates the basement membrane. Scale bar: 50 µm. (E) Cell number per area (2000 µm2) of control (37.6±4.59 cells, n = 3) and Sfrp1−/− Sfrp2−/− Sfrp5+/− (48.5±7.43 cells, n = 3) fore-stomach epithelium. (F) Frequency of multi-nuclei along the AB axis in the greater curvature epithelium of control and Sfrps-deficient fore-stomachs (4.37±2.01% of 343 control epithelial cells, n = 3; 4.11±1.30% of 438 Sfrp1−/− Sfrp2−/− Sfrp5+/− epithelial cells, n = 3). (2.28 MB TIF) [file pgen.1000427.s004.tif]

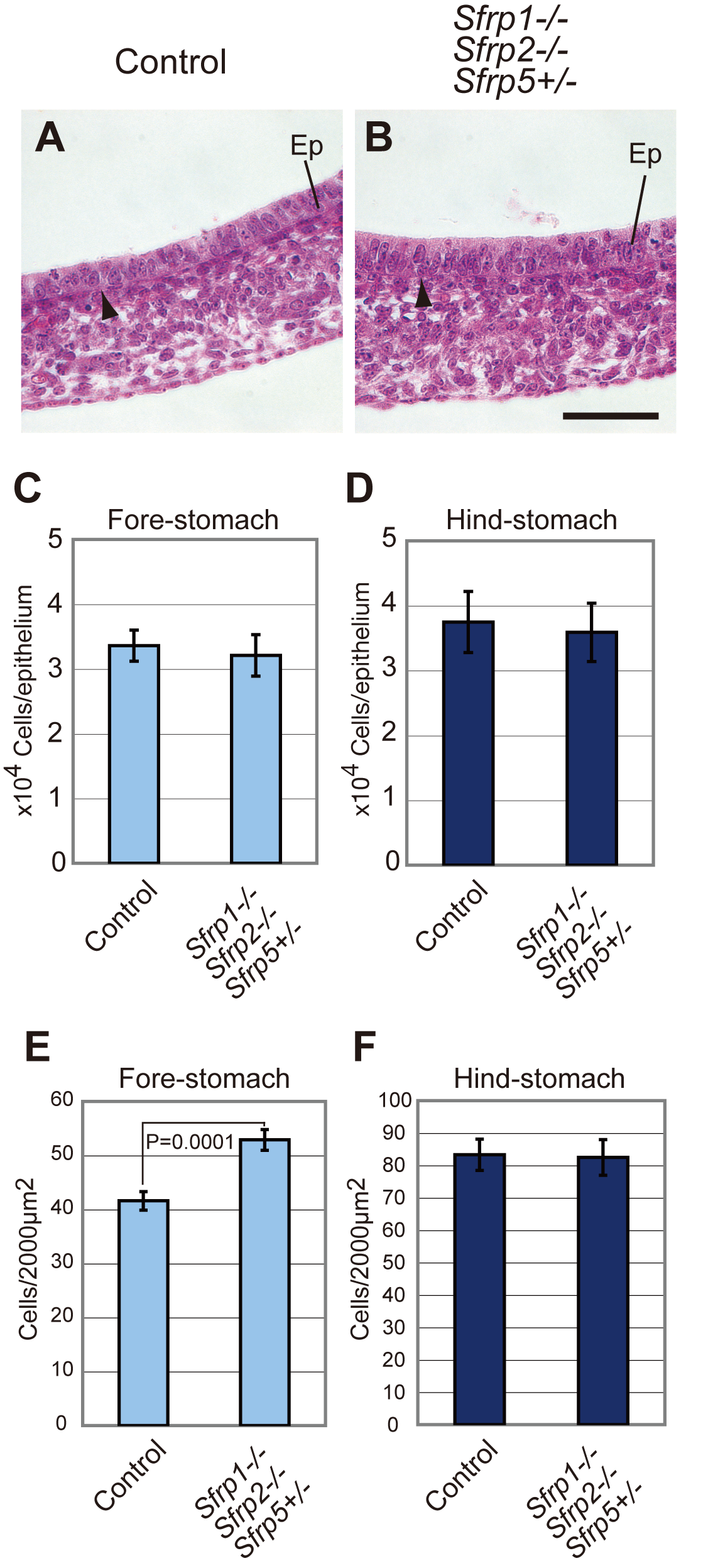

Supplement: Figure S5 — Sfrp1−/− Sfrp2−/− Sfrp5+/− fore-stomach epithelium at E13.5. (A, B) The greater curvature epithelium of control (A) and Sfrp1−/− Sfrp2−/− Sfrp5+/− (B) fore-stomachs. Ep, epithelium. The arrowhead indicates the basement membrane. Scale bar: 50 µm. (C, D) Total cell number in the epithelium is unaltered in Sfrp1−/− Sfrp2−/− Sfrp5+/− fore-stomach (C; 3.21±0.32×10^4^ cells, n = 3) as well as in Sfrp1−/− Sfrp2−/− Sfrp5+/− hind-stomach (D; 3.59±0.45×10^4^ cells, n = 3) in comparison to the control fore- (3.36±0.24×10^4^ cells, n = 3) and hind-stomach (3.75±0.47×10^4^ cells, n = 3). (E, F) Epithelial cell number per area (2000 µm2) increased approximately 27% in the greater curvature of Sfrp1−/− Sfrp2−/− Sfrp5+/− fore-stomach (52.9±1.92 cells) versus that of control (E; 41.6±1.73 cells, n = 4); however, no difference was observed in the hind-stomachs derived from control (83.3±4.8 cells) and Sfrp1−/− Sfrp2−/− Sfrp5+/− (82.5±5.5 cells) embryos (F; n = 4). (1.53 MB TIF) [file pgen.1000427.s005.tif]

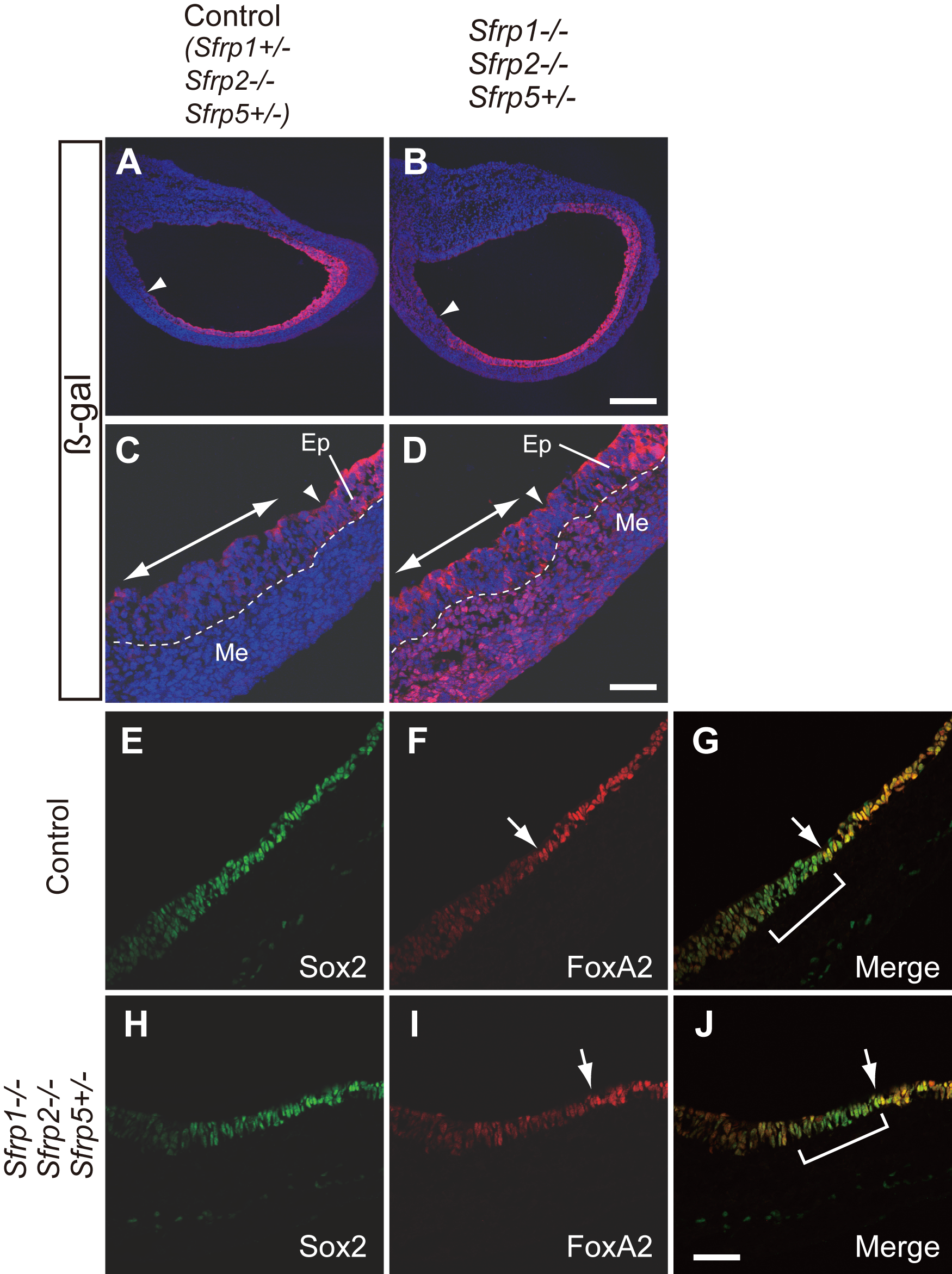

Supplement: Figure S6 — The Wnt/β-catenin pathway in Sfrps-deficient stomach epithelium. (A, B) TOPGAL activity visualized by anti-β-galactosidase antibody staining indicates higher canonical Wnt/β-catenin signaling activity in control (A) and Sfrp1−/− Sfrp2−/− Sfrp5+/− (B) fore-stomachs at E13.5. Scale bar: 200 µm. (C, D) Reporter activity indicates slightly enhanced canonical Wnt/β-catenin signaling in Sfrp1−/− Sfrp2−/− Sfrp5+/− hind-stomach epithelium (D) in comparison with the control (C). Scale bar: 50 µm. Note that β-galactosidase derived from the TOPGAL reporter exhibits cytoplasmic localization in the epithelium (Ep), whereas β-galactosidase derived from the Sfrp1 knock-in locus displays nuclear localization in the mesenchyme (Me). The arrowhead identifies a boundary between fore- and hind-stomach epithelium as determined by epithelial morphology. (E–J) Protein distribution of FoxA2 and Sox2 is not significantly altered in the junction between the fore- and hind-stomach in Sfrp1−/− Sfrp2−/− Sfrp5+/− embryos in comparison to control embryos at E13.5. Higher FoxA2 expression is found in fore-stomach epithelium; in contrast, the expression weakens at the junction of the fore- and hind-stomach epithelium (arrow). Sox2 expression in fore-stomach epithelium is gradually reduced in the hind-stomach epithelium (bracket). Scale bar: 50 µm. (2.83 MB TIF) [file pgen.1000427.s006.tif]

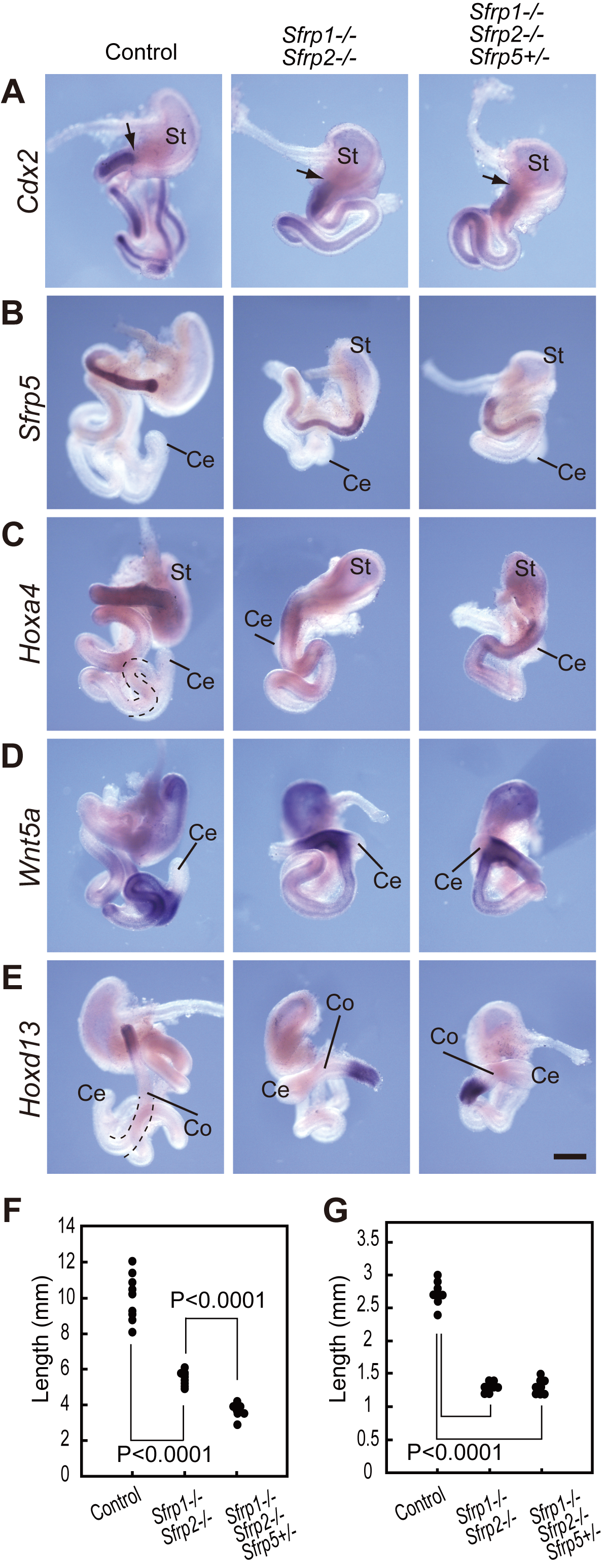

Supplement: Figure S7 — Regionalization of the intestine in Sfrp1−/− Sfrp2−/− and Sfrp1−/− Sfrp2−/− Sfrp5+/− embryos at E13.5. (A–E) The intestines of Sfrp1−/− Sfrp2−/− and Sfrp1−/− Sfrp2−/− Sfrp5+/− embryos are regionalized along the cephalocaudal axis, as suggested by the expressions of Cdx2 (A), Sfrp5 (B), Hoxa4 (C), Wnt5a (D) and Hoxd13 (E). Ce, cecum; Co, colon; St, stomach. Scale bar: 500 µm. (F) The length of the small intestine was reduced in Sfrp1−/− Sfrp2−/− embryos (5.5±0.39 mm; n = 9; P<0.0001) relative to control small intestine at E13.5 (10.1±1.32 mm; n = 9). The reduction in length was enhanced upon introduction of an Sfrp5 mutant allele (3.7±0.37 mm; n = 9; P<0.0001). (G) The length of the rostral large intestine coinciding with the Hoxd13-negative region is shortened in Sfrp1−/− Sfrp2−/− (1.30±0.076 mm; n = 8; P<0.0001) and Sfrp1−/− Sfrp2−/− Sfrp5+/− (1.31±0.11 mm; n = 8; P<0.0001) embryos in comparison with controls (2.73±0.18 mm; n = 9) at E13.5. (3.81 MB TIF) [file pgen.1000427.s007.tif]

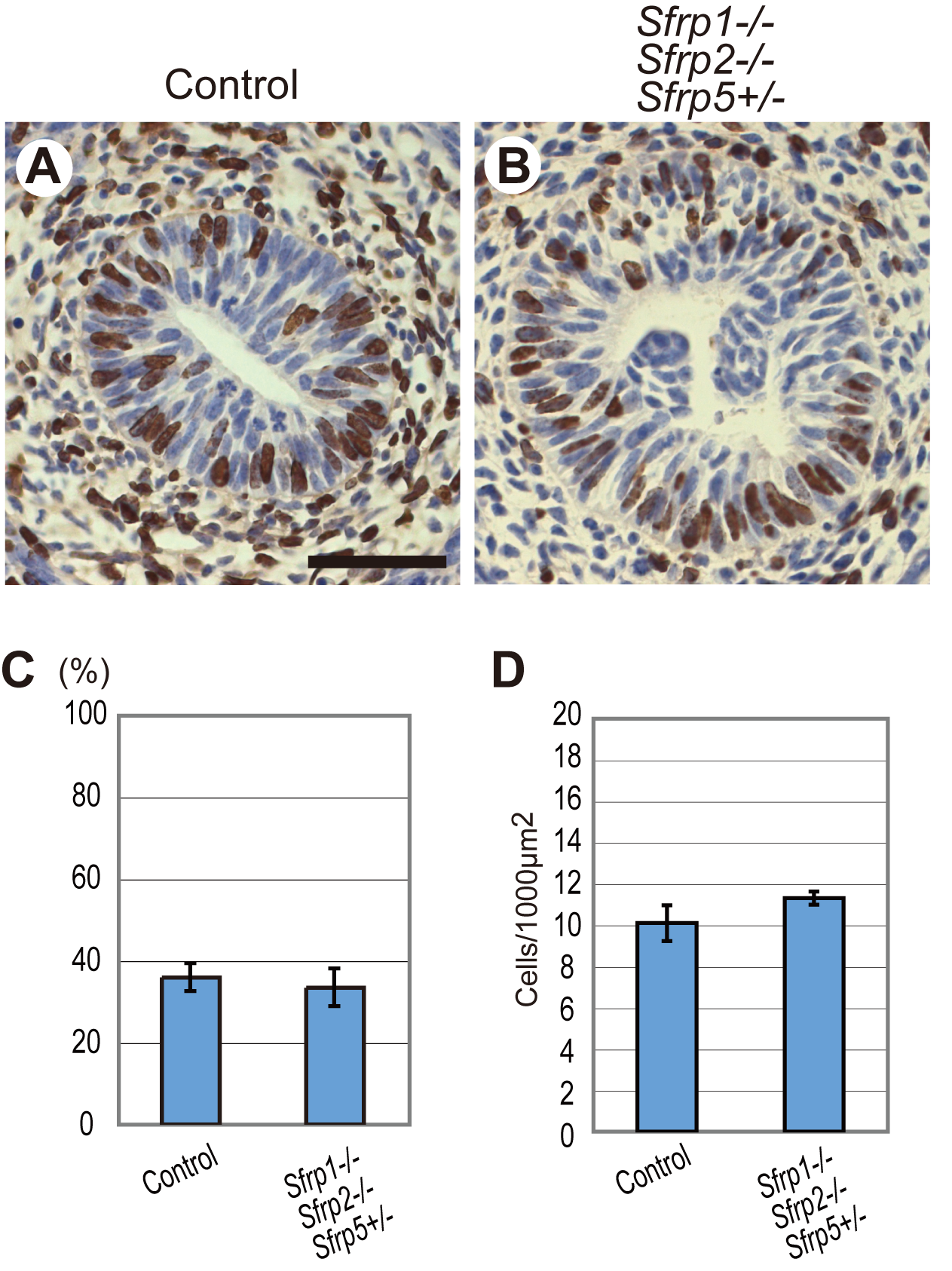

Supplement: Figure S8 — BrdU incorporation assay in Sfrps-deficient small intestine. (A–C) Cell proliferation ratios increased in neither the epithelial cell clump (A, B) nor the entire epithelium (C) in the Sfrp1−/− Sfrp2−/− Sfrp5+/− small intestine in comparison with control small intestine at E13.5. Scale bar: 50 µm. (D) Cell density was not significantly increased in the epithelium of Sfrp1−/− Sfrp2−/− Sfrp5+/− small intestine (11.3±0.32 cells/1000 µm2, n = 3) in comparison with control epithelium (10.1±0.87 cells/1000 µm2, n = 3) at E13.5. (1.78 MB TIF) [file pgen.1000427.s008.tif]

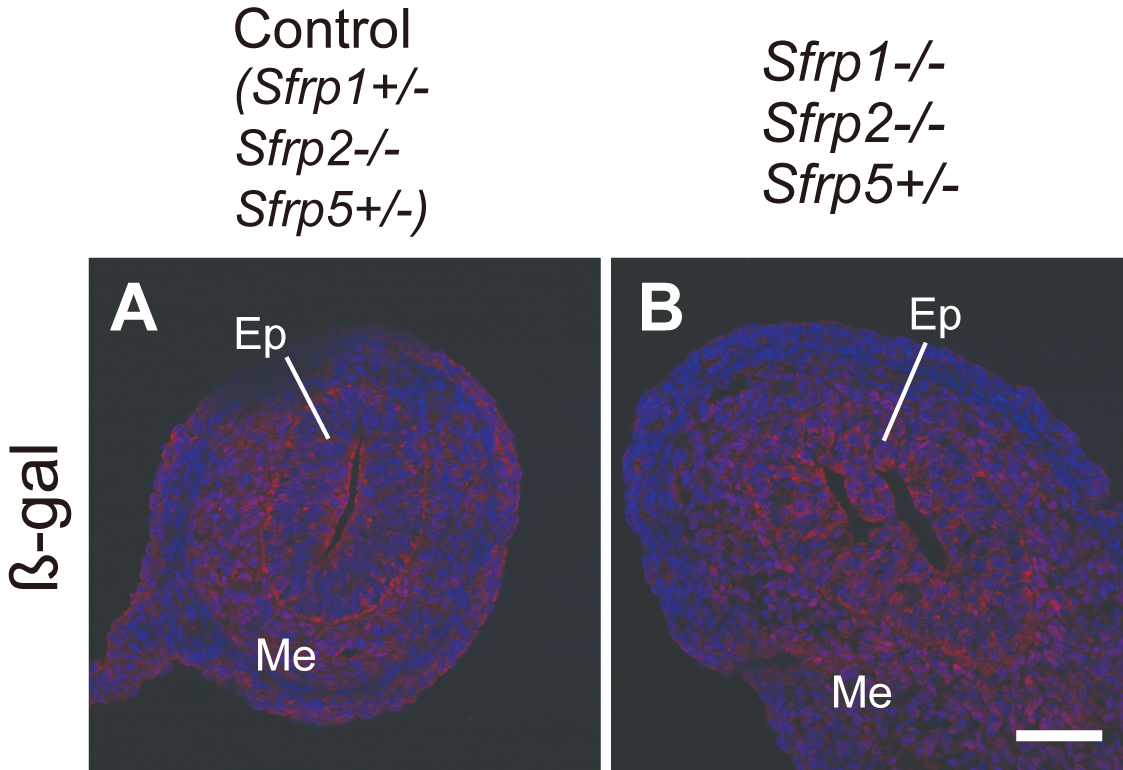

Supplement: Figure S9 — The Wnt/ß-catenin pathway is not enhanced in Sfrp1−/− Sfrp2−/− Sfrp5+/− small intestine. (A, B) TOPGAL activity visualized by anti-β-galactosidase antibody staining indicates that Wnt/β-catenin signaling activity is not enhanced in Sfrp1−/− Sfrp2−/− Sfrp5+/− small intestine epithelium at E13.5. Scale bar: 50 µm. (1.00 MB TIF) [file pgen.1000427.s009.tif]
